# Maternal Levels of Cytokines in Early Pregnancy and Risk of Autism Spectrum Disorders in Offspring

**DOI:** 10.3389/fpubh.2022.917563

**Published:** 2022-05-31

**Authors:** Martin Brynge, Renee M. Gardner, Hugo Sjöqvist, Brian K. Lee, Christina Dalman, Håkan Karlsson

**Affiliations:** ^1^Department of Global Public Health, Karolinska Institutet, Stockholm, Sweden; ^2^Department of Epidemiology and Biostatistics, Drexel University School of Public Health, Philadelphia, PA, United States; ^3^A.J. Drexel Autism Institute, Philadelphia, PA, United States; ^4^Centre for Epidemiology and Community Medicine, Region Stockholm, Stockholm, Sweden; ^5^Department of Neuroscience, Karolinska Institutet, Stockholm, Sweden

**Keywords:** autism, cytokines, pregnancy, ADHD, intellectual disability

## Abstract

Previous studies indicate a role of immune disturbances during early development in the etiology of autism spectrum disorders (ASD). Any potential disturbances during fetal development are best addressed by prospective evaluation of maternal markers of inflammation. Previous studies have investigated maternal cytokines, a group of powerful effectors of the immune system, with inconsistent results. In this study, we aimed to clarify the relationship between maternal cytokines and ASD by evaluating levels of 17 cytokines in first trimester maternal serum samples, from 318 mothers to ASD-cases and 429 mothers to ASD-unaffected controls, nested within the register-based Stockholm Youth Cohort. Overall, we observed no consistent associations between levels of maternal cytokines and ASD. While we observed a number of individual associations, the patterns varied across the diagnostic sub-groups. Levels above the 90th percentile of IL-1β (OR = 2.31, 95% CI 1.16–4.60), IL-7 (OR = 2.28, 95% CI 1.20–4.33), IL-13 (OR = 2.42, 95% CI 1.29–4.55), and MCP-1 (OR = 2.09, 95% CI 1.03–4.24) were associated with increased odds of ASD with co-occurring intellectual disability (ID), whereas GMCSF (OR = 2.06, 95% CI 1.03–4.11) and TNF-α (OR = 2.31, 95% CI 1.18–4.50) were associated with increased odds of ASD with ADHD but none survived correction for multiple comparisons. Also, none of the measured maternal cytokines were associated with ASD without co-occurring ID or ADHD. Implementing a data-driven approach using machine learning (Random Forest's Variable Importance measurement), we found no evidence to suggest that adding these cytokines and other markers of maternal immunity, to register-based maternal factors (e.g., psychiatric history) improves prediction of ASD. In summary, we found no robust evidence of an association between maternal immune markers during early pregnancy and ASD.

## Introduction

Autism Spectrum Disorder (ASD) is a heterogeneous group of neurodevelopmental disorders characterized by social impairments, restricted and repetitive patterns of behavior, and atypical sensory responses (1). The diagnosis is usually made in childhood, indicating the overall importance of causal factors acting during early development (2). The liability for ASD is dominated by genetic variation at the population level, although evidence also supports significant influences from environmental factors (3). The relative contribution of genetic and environmental factors to the etiology of ASD may vary according to commonly co-occurring diagnoses such as attention deficit/hyperactivity disorder (ADHD) and intellectual disability (ID) (4).

Several factors related to the prenatal environment have been linked to ASD in observational studies (5). A number of these environmental exposures during pregnancy, such as maternal infections (6), air pollution (7), and high BMI (8), are associated with altered systemic levels of cytokines and immune function (9–11). The maternal immune system has several functions during pregnancy that are important for normal fetal development (12). It has been hypothesized that perturbations of immune signaling pathways during pregnancy alter normal trajectories of pregnancy and fetal development, increasing the risk of ASD (13). Indeed, experimental murine models strongly suggest that activation of the maternal immune system during gestation can cause behavioral abnormalities in the offspring, including autism-related phenotypes, though the validity of such animal models remains to be established (14, 15).

Cytokines are a group of powerful regulators of the immune system, secreted mainly by immune cells such as tissue resident macrophages and lymphocytes, but also by other cell types, including endothelial cells and adipocytes. Cytokines play important regulatory roles at different stages of pregnancy, e.g., embryo implantation and placental development (12, 16), and maternal serum concentrations may vary considerably over the course of pregnancy (17). Nearly all known cytokines, including the pro-inflammatory IL-6, IL-1β and TNF-α, are also produced by the placenta (16). Various pregnancy complications, including gestational diabetes and maternal infections, are associated with dysregulated release of placental cytokines (18). A change in placental cytokine secretion can be caused by the actions of maternal cytokines on placental cells, and placental immune activation may thus transfer effects of a maternal immune disturbance to the fetus (19, 20). Moreover, some cytokines have the ability to reach the fetal circulation via direct transfer across the placental barrier (21). Thus, a maternal immune perturbation may be communicated across the placenta even without the direct transmission of the underlying causative agent, e.g., an infection. An altered cytokine profile in the fetal compartment may interfere with neurodevelopmental processes, such as neuronal migration and differentiation, glial cell activation, and synaptic pruning which all rely on immune signaling molecules, to affect the normal developmental trajectory of the fetal brain (22).

Several previous studies have investigated maternal cytokines during pregnancy related to offspring risk of ASD, all use samples collected in mid-pregnancy (23–26). The results are inconsistent, with significant findings failing to replicate across studies, for example, both elevated and decreased levels of IL-4 have been reported among mothers to ASD-cases (23, 26). However, there are substantial differences related to design and methodology, and only one of the studies used clinically evaluated cases in a large population-based sample. No studies to date have measured cytokines at a time-point other than the second or third trimesters.

Cytokines and other immune markers measured in early life may be useful as biomarkers for ASD, with potential implications for early detection and development of novel preventive strategies (27–29). In this study, we investigate the relationship between maternal immune status and ASD by measuring 17 cytokines in archived first trimester maternal serum samples, using different analytical strategies. First, we estimate the associations between individual cytokines and ASD. Second, we conduct a principal component analysis (PCA) to integrate the collective information from the entire range of immune markers measured, combining data on the cytokines with previous measurements of maternal acute phase proteins (APP) (30). We use the derived principal components to investigate if the ASD-cases and controls can be separated based on their immune marker profiles. Finally, we employ a random forest variable importance analysis to investigate if information on maternal immune markers in the first trimester, collectively, can improve the prediction of ASD, when added to other known predictors of ASD, such as maternal age, psychiatric illness and sex of the child.

## Materials and Methods

### Study Population

This study is nested within the Stockholm Youth Cohort (SYC), a population-based cohort including all individuals born in Sweden and resident in Stockholm County for ≥4 years (31) and covering all pathways to psychiatric care and habilitation services within Stockholm County for the purposes of outcome ascertainment.

The study design and collection of biological samples have been described in detail previously (30). Briefly, our source population consists of children within SYC born 1996–2000 (*n* = 98,597). We collected archived Neonatal Dried Blood Spots (NDBS) from a national biobank at Karolinska University Hospital, Solna, from nearly all children with a diagnosis of ASD (*n* = 1,407) and a random sample of ASD-unaffected control individuals (*n* = 1,847) (32). For a subset of the children with available NDBS, we also collected corresponding archived serum samples from mothers to ASD cases (*n* = 430) and mothers to controls (*n* = 549). Ethics approval was obtained by the Stockholm regional review board (DNR 2010/1185-31/5). Individual consent was not required for this anonymized register-based study.

### Case Ascertainment

National and regional registers were used for the ascertainment of ASD, ID and ADHD, covering all pathways to care and diagnosis in Stockholm County. The case-finding procedure has been described in detail previously (31, 32). ASD was stratified by co-occurrence of ID and ADHD: ASD only (ASD without ID or ADHD), ASD with ID, and ASD with ADHD. The ASD with ID group also included individuals with co-morbid ID and ADHD.

### Laboratory Analysis

Archived maternal serum samples initially drawn as part of an antenatal screening program for maternal infections, were collected from regional biobanks at Karolinska University Hospital, Solna and Huddinge. Samples are usually, but not always, drawn at the first antenatal visit, near the end of the first trimester [median = 10.9 gestational weeks, interquartile range (IQR) 9.3–12.7]. In order to compare samples from the same stage of development, the samples were restricted to those drawn within the first trimester, resulting in a final study population of 318 ASD and 429 control mothers for the primary statistical analyses (30). After thawing on ice, samples were diluted 1:4 and analyzed for Interleukin (IL)-1β, IL-2, IL-4, IL-5, IL-6, IL-7, IL-8, IL-10, IL-12, IL-13, IL-17, Granulocyte Colony-Stimulating Factor (GCSF), Granulocyte Monocyte Colony-Stimulating Factor (GMCSF), Interferon (IFN)-γ, Monocyte Chemoattractant Protein 1 (MCP-1), Macrophage Inflammatory Protein 1b (MIP-1β), Tumor Necrosis Factor-α (TNF-α) using the Bio-Plex Pro Human Cytokine 17-plex Assay (Bio-Rad, Hercules, CA, USA). Samples were applied randomly to 13 multiplex 96-well assay plates and analyzed using a premixed, multiplex panel on the Bio-Plex 200 System (Bio-Rad). There were two types of imputed values that resulted from samples with concentrations near or beyond the limits of quantitation (see Supplementary Table 1). Concentrations near the lower limit of quantitation (LLOQ) are assigned an imputed value by the BioPlex Manager software but are also marked as potentially uncertain estimates. In these cases, we used the value estimated by the software in our analyses but classified the values as imputed. Concentrations below the LLOQ (with no value estimated by the software) were assigned a value of LLOQ/√2, whereas values above the upper limit of quantitation (ULOQ) were assigned a value of ULOQ×1.1.

### Covariates

Based on previous associations with ASD, and a plausible relationship with cytokines, we considered the following covariates as potential confounders: maternal age (33), psychiatric history (34, 35), BMI (8), region of birth (36), education (34), and smoking at first antenatal visit (37), sex of fetus (31), birth order, family income (38), and gestational week and season at serum sample (39). Covariate data were extracted from the Medical Birth Register, the National Patient Register and the Integrated Database for Labor Market Research.

### Statistical Analysis

Data management and analyses were performed using Stata (v14.1) with external package xbrcspline (40), and R Statistical Software (v4.1.0), with external packages “randomForest,” “ggplot2” and “factoextra” (41–44).

Due to skewed distributions, the concentrations of cytokines were log2-transformed (Supplementary Figures 1A,B). Plate specific standardized z-scores were created (Supplementary Figure 1C) to control for assay plate technical variation.

In our previous study of maternal APP in these same samples, the exposure variables were categorized into tertiles (30). In the present study, 11 of the total 17 cytokines had tertile cut-points within the non-detectable range. Since this strategy would exclude most of our analytes, we instead considered a dichotomous categorization based on the 90th percentile (≥90th percentile; <90th percentile), in order to make use of all data, and to better capture variation at the high end of the distribution. Similar analytical strategies have been used in previous studies of cytokines and ASD in archived samples (28, 45).

We tested the associations of covariates and cytokines by using a univariate linear regression model estimating mean cytokine z-scores over the categories of covariates, followed by a joint Wald-test of the overall of association between the cytokine and the covariate. Covariates were included in the models if they were even weakly associated (*p* < 0.2) with any of the outcomes and at least one of the cytokines among controls. For the cytokines with a low proportion of samples falling within the limits of detection and thus a high proportion (>70%) of imputed values (see “Laboratory Analysis” above, Supplementary Table 1), we instead evaluated associations with covariates by applying a chi-2 test using the dichotomous cytokine variables (based on the 90th percentile).

In the categorical analyses, we used logistic regression models to estimate the odds of ASD associated with dichotomized levels of each cytokine (based on the 90th percentile), with the lowest category (≥90th percentile) as the referent level. The analyses were repeated after stratifying on the co-occurrence of ID and ADHD. After dichotomizing the sample at the 90th percentile based on the distribution of the measured cytokines, we calculated that we would be able to detect an odds ratio of 1.92 at 80% power given the size of our study at an α level of 0.05. Because numerous statistical comparisons were made, we considered two approaches to correct for multiple comparisons. For the Bonferroni correction, the specified α (*p* = 0.05) is divided by the number of independent tests conducted. We calculated a Bonferroni-corrected *p*-value considering either 17 tests (if one considers the 17 cytokines and the main outcome of any ASD diagnosis; *p* < 0.0029) or 68 tests (if one considers the 17 cytokines across the main outcome and the three mutually exclusive outcomes; *p* < 0.0007). Bonferroni correction is considered to be highly conservative (46), and it is arguable that the tests in this case (of correlated cytokine values across related outcomes) are truly independent. Because of these issues, we also considered the Benjamini-Hochberg procedure to control the False Discovery Rate (FDR). We set the FDR, defined as the proportion of all “positive” findings (where the null hypothesis was rejected, *p* < 0.05) which are false positives (Type I errors in which the null hypothesis was incorrectly rejected), to levels of 5, 10, and 25%, respectively, and considered 68 tests.

In the continuous analyses, we used restricted cubic spline models with three knots, followed by a Wald-test of all spline variables to test for an association between the cytokine and the outcome of ASD. Only analytes with <70% imputed values were included in these analyses.

The relationship between the maternal immune status and ASD is likely more complex than what can be captured by investigating one-by-one associations with individual cytokines. However, using the cytokines collectively in the same regression model is problematic, due to their high correlation/dependency and the risk of model overfitting. To achieve a more integrated view of the maternal immune status and its relationship with children's risk of ASD, and to simultaneously deal with the multiple correlated analytes, we used Principal Component Analysis (PCA) to re-construct the covariation between all the biomarkers into independent components. To explore the full range of available immune markers, we combined the cytokines measurements with the previously reported first trimester maternal APP (30), measured in the same cohort of pregnant women in the same serum samples. We used the extracted principal components (selecting those that explained >5% of variation) to examine any potential relation with ASD and the stratified outcomes, using cubic spline models with 4 knots to account for potentially non-linear relationships.

Finally, we used the Random Forest Classifier to assess the predictive performance of the biomarker measurements in comparison to other covariates and to compute the variable importance on the full sample size (*n* = 747). We used the Mean Decrease Accuracy measurement for the variable importance, which employs an algorithm to replace a variable with a randomly generated number and examine the decrease in the new prediction accuracy. To assess the predictive performance of the respective models, we randomly divided the data into a training set (2/3 of the sample) and a test set (1/3 of the sample) and ascertained the overall accuracy. We explored the performance of different sub-sets of our data by using as predictors: (a) only the non-biomarker (i.e., register-based) covariates used in the fully adjusted regression models (sex of fetus; family income quintile; maternal education, BMI, psychiatric history, region of origin, and age), (b) only the six largest PCA-components, (c) the six largest PCA-components together with the non-biomarker covariates. Because random forest models can in principle deal with a very large number of correlated covariates and therefore does not require dimension reduction techniques (e.g., extraction of principle components from PCA), we alternatively fit random forest models including as predictors: (d) the original cytokine and APP z-score variables only, and (e) the original cytokine and APP z-score variables together with the register-based covariates. We repeated these steps 1,000 times with randomized training and test sets and estimated the mean accuracy together with the 2.5th and 97.5th percentile value (i.e., 95% pseudo-bootstrap confidence interval).

### Sensitivity Analysis

In the main analyses, the samples were restricted to those drawn in the first trimester of pregnancy, in order to reduce variation in cytokines related to gestational age and improve interpretability. However, this resulted in the exclusion of 232 individuals for whom maternal sera were available. As a sensitivity analysis, we therefore repeated the categorical analysis (using the 90th percentile cutoff for the cytokines) including all available serum samples and additionally adjusted for gestational week at sampling (<10, 10–13, and >13 weeks). We also repeated the PCA using information from all available serum samples (*n* = 979) and evaluated any associations with ASD.

## Results

### Association of Covariates With ASD-Case Status

Compared to unaffected controls, ASD-cases were more likely to be male. Mothers to ASD-cases were more likely to be below 30 or above 40 years of age, have a history of psychiatric disease, be born outside the Nordic region, and have a lower socioeconomic status (Table 1).

**Table 1:** Characteristics of individuals diagnosed with ASD and unaffected individuals in the study sample^a^.

	**Unaffected** **(*n* = 429)**	**ASD** **(*n* = 318)**	***p*-value^b^**	**ASD only** **(*n* = 100)**	**ASD with ID** **(*n* = 101)**	**ASD with ADHD** **(*n* = 117)**	***p*-value^c^**
**Sex**							
Female	204 (47.6%)	69 (21.7%)	<0.001	19 (19.0%)	27 (26.7%)	23 (19.7%)	<0.001
Male	225 (52.4%)	249 (78.3%)		81 (81.0%)	74 (73.3%)	94 (80.3%)	
**Birth order**							
1st born	187 (43.6%)	164 (51.6%)	0.090	57 (57.0%)	40 (39.6%)	67 (57.3%)	0.031
2nd born	167 (38.9%)	109 (34.3%)		30 (30.0%)	45 (44.6%)	34 (29.1%)	
3rd or higher	75 (17.5%)	45 (14.2%)		13 (13.0%)	16 (15.8%)	16 (13.7%)	
**Maternal age (years)**							
<25	43 (10.0%)	38 (11.9%)	0.041	8 (8.0%)	12 (11.9%)	18 (15.4%)	0.054
25–29	108 (25.2%)	101 (31.8%)		29 (29.0%)	27 (26.7%)	45 (38.5%)	
30–34	178 (41.5%)	101 (31.8%)		34 (34.0%)	37 (36.6%)	30 (25.6%)	
35–39	89 (20.7%)	64 (20.1%)		24 (24.0%)	19 (18.8%)	21 (17.9%)	
3 40	11 (2.6%)	14 (4.4%)		5 (5.0%)	6 (5.9%)	3 (2.6%)	
**Maternal psychiatric history**							
No	289 (67.4%)	162 (50.9%)	<0.001	50 (50.0%)	60 (59.4%)	52 (44.4%)	<0.001
Yes	140 (32.6%)	156 (49.1%)		50 (50.0%)	41 (40.6%)	65 (55.6%)	
**Maternal BMI**							
Underweight	8 (1.9%)	7 (2.2%)	0.057	2 (2.0%)	3 (3.0%)	2 (1.7%)	0.005
Normal	213 (49.7%)	126 (39.6%)		49 (49.0%)	43 (42.6%)	34 (29.1%)	
Overweight	59 (13.8%)	54 (17.0%)		11 (11.0%)	18 (17.8%)	25 (21.4%)	
Obese	16 (3.7%)	21 (6.6%)		2 (2.0%)	6 (5.9%)	13 (11.1%)	
Missing	133 (31.0%)	110 (34.6%)		36 (36.0%)	31 (30.7%)	43 (36.8%)	
**Maternal region of birth**							
Africa	18 (4.2%)	16 (5.0%)	0.89	3 (3.0%)	12 (11.9%)	1 (0.9%)	<0.001
Asia	34 (7.9%)	27 (8.5%)		8 (8.0%)	18 (17.8%)	1 (0.9%)	
Nordic	349 (81.4%)	250 (78.6%)		83 (83.0%)	61 (60.4%)	106 (90.6%)	
Other	15 (3.5%)	12 (3.8%)		4 (4.0%)	4 (4.0%)	4 (3.4%)	
Other Europe	13 (3.0%)	13 (4.1%)		2 (2.0%)	6 (5.9%)	5 (4.3%)	
**Family income quintile**							
1st (lowest)	41 (9.6%)	39 (12.3%)	0.006	8 (8.0%)	22 (21.8%)	9 (7.7%)	<0.001
2nd	75 (17.5%)	81 (25.5%)		20 (20.0%)	28 (27.7%)	33 (28.2%)	
3rd	90 (21.0%)	64 (20.1%)		22 (22.0%)	17 (16.8%)	25 (21.4%)	
4th	100 (23.3%)	75 (23.6%)		18 (18.0%)	23 (22.8%)	34 (29.1%)	
5th	123 (28.7%)	59 (18.6%)		32 (32.0%)	11 (10.9%)	16 (13.7%)	
**Maternal education at birth**							
<9 years	55 (12.8%)	42 (13.2%)	0.22	10 (10.0%)	13 (12.9%)	19 (16.2%)	0.14
9–12 years	179 (41.7%)	151 (47.5%)		41 (41.0%)	52 (51.5%)	58 (49.6%)	
>12 years	194 (45.2%)	124 (39.0%)		49 (49.0%)	35 (34.7%)	40 (34.2%)	
Missing	1 (0.2%)	1 (0.3%)			1 (1.0%)		
**Gestational week at serum sample**							
<10 weeks	203 (47.3%)	160 (50.3%)	0.42	52 (52.0%)	48 (47.5%)	60 (51.3%)	0.77
10–13 weeks	226 (52.7%)	158 (49.7%)		48 (48.0%)	53 (52.5%)	57 (48.7%)	
**Serum sampling quarter**							
1 January−31 March	126 (29.4%)	98 (30.8%)	0.33	30 (30.0%)	35 (34.7%)	33 (28.2%)	0.54
1 April−30 June	101 (23.5%)	90 (28.3%)		34 (34.0%)	24 (23.8%)	32 (27.4%)	
1 July−30 September	96 (22.4%)	64 (20.1%)		17 (17.0%)	23 (22.8%)	24 (20.5%)	
1 October−31 December	106 (24.7%)	66 (20.8%)		19 (19.0%)	19 (18.8%)	28 (23.9%)	
**Smoking at first antenatal visit**							
No	297 (69.2%)	220 (69.2%)	1.00	67 (67.0%)	74 (73.3%)	79 (67.5%)	0.31
Yes	27 (6.3%)	20 (6.3%)		3 (3.0%)	5 (5.0%)	12 (10.3%)	
Missing	105 (24.5%)	78 (24.5%)		30 (30.0%)	22 (21.8%)	26 (22.2%)	

a *The characteristics of the same population has been reported previously (30), https://creativecommons.org/licenses/by/4.0/*.

b *Pearson's chi-squared test was used for categorical variables, comparing the frequency distributions among unaffected individuals to the distributions among all ASD-affected individuals. Kruskal-Wallis tests were used for continuous variables, as the distributions of the APP concentrations were strongly skewed*.

c *Pearson's chi-squared test was used for categorical variables, comparing the frequency distributions among unaffected individuals to the distribution among the stratified ASD outcome groups. Kruskal-Wallis tests were used for continuous variables, as the distributions of the APP concentrations were strongly skewed*.

*ASD, autism spectrum disorders; ADHD, attention-deficit/hyperactivity disorder; ID, intellectual disability; BMI, body mass index; IQR, interquartile range*.

### Quality Control Statistics for Cytokines

The proportion of imputed values (due to observations below or near the LLOQ) varied from 0% (MIP-1β) to 93.3% (IL-5) (Supplementary Table 1). Seven cytokines had a proportion of imputed values >70%. The average inter- and intra-assay coefficients of variation (CV) of manufacturer-provided controls were 21.2% (range 15.1–34.9%) and 6.1 (range 2.5–9.2%), respectively (Supplementary Table 1). Among unaffected controls, we observed moderate to high degrees of pairwise correlations between the different cytokines (Supplementary Figure 2A). Overall, TNF-α, IL-6 and IL-4 showed the highest pairwise correlations with the other cytokines. Similar patterns of correlation were seen when only values within the detectable range were included (Supplementary Figure 2B).

### Association of Cytokines With Covariates

We observed a significant (*p* < 0.05) linear relationship between IL-8, IL-17, GCSF, MCP-1 and MIP-1β, and gestational age, using all available samples (*n* = 979, Supplementary Table 2). Levels of IL-8, IL-17, MCP-1, and MIP-1β tended to decrease, whereas GCSF tended to increase, over the course of pregnancy. A linear relationship between IL-6 and gestational age was observed in samples restricted to the first trimester and an inverse linear association between gestational age and MIP-1β was observed in samples restricted to the 2nd and 3rd trimesters.

**Figure 1:**
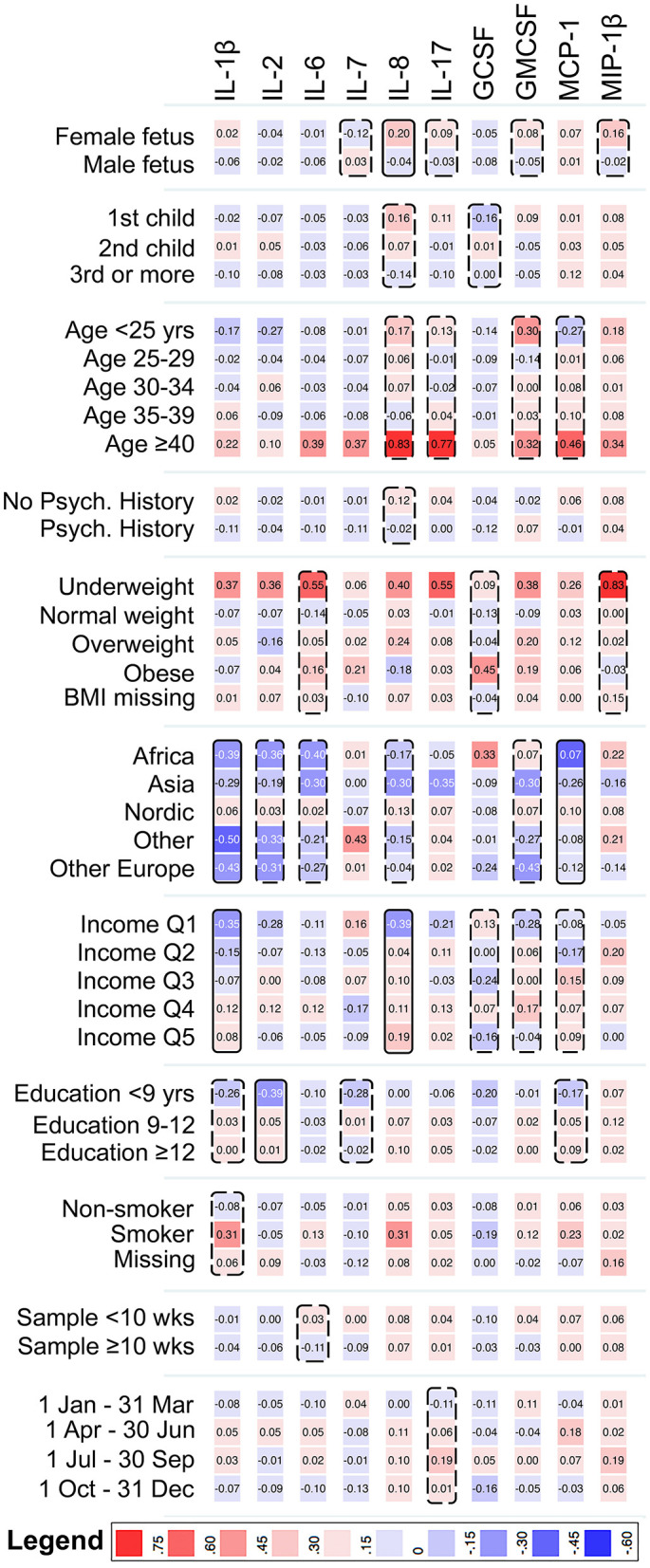
Heat map showing the mean z-score of cytokines with <70% imputed values, by categories of the covariates, among mothers to 429 unaffected individuals in the cohort. Solid boxes indicate that the cytokine is associated with the covariate at *p* < 0.05. Dashed boxes indicate that the cytokine is associated with the covariate at *p* < 0.20. IL-1β, Interleukin-1β; IL-2, Interleukin-2; IL-6, Interleukin-6; IL-7, Interleukin-7; IL-8, Interleukin-8; IL-17, Interleukin-17; GCSF, Granulocyte Colony-Stimulating Factor; GMCSF, Granulocyte Monocyte Colony-Stimulating Factor; MCP-1, Monocyte Chemoattractant Protein 1; MIP-1β, Macrophage Inflammatory Protein 1β.

Among mothers to controls, sex of fetus, maternal region of birth, parental income, and maternal education, were associated with one, or more, of the cytokines at our pre-defined level to consider as a potential confounder (*p* < 0.2; Figure 1). The strongest associations were observed for maternal region of birth with IL-1β and MCP-1, parental income with IL-1β and IL-8, and maternal education with IL-2 (*p* < 0.05). Among mothers to ASD-cases, some of the associations overlapped with the control group (maternal region of birth with MCP-1, and income with IL-8), whereas others were unique for the ASD-case group (sex of fetus with MIP-1β, maternal smoking with IL-7, and sampling season with GCSF) (Supplementary Figure 3).

For the cytokines with a low proportion of samples falling within the limits of detection, all the considered covariates except maternal region of birth and sampling week were associated (*p* < 0.2) with at least one of the cytokines (testing the proportion of samples in the highest decile), among mothers to controls (Supplementary Figure 4). The specific patterns of association differed by case status (Supplementary Figure 5). For example, BMI was associated with TNF-α among mothers in the control group, but not among mothers to ASD-cases.

The covariates that met the a priori criteria for inclusion in the adjusted regression models were: sex of fetus; family income quintile; maternal education, BMI, psychiatric history, region of origin, and age. Maternal education was excluded from the models due to the high degree of correlation with parental income.

### Association of Cytokines With Odds of ASD

There were no significant differences in median concentrations of cytokines with low proportion of imputed <70% imputed values between ASD-cases and unaffected controls (Table 2; Supplementary Figure 6). For those cytokines with >70% of imputed values, the proportion of observations in the highest decile were significantly (*p* < 0.05) larger for IL-10 in cases compared to controls (Table 2). In the unadjusted categorical regression analysis, using the 90th percentile among controls as cut-off, significant associations (*p* < 0.05) were observed between IL-7, IL-10 and IL-13, and odds of ASD with ID, and between TNF-α and ASD with ADHD (Supplementary Table 3). In the adjusted regression models (Table 3), levels of the pro-inflammatory cytokines IL-1β (OR = 1.71, *p* = 0.030), IL-6 (OR = 1.68, *p* = 0.037), and TNF-α (OR = 1.79, *p* = 0.020), and the inhibitory cytokine IL-10 (OR = 1.74, *p* = 0.024) at or above the 90th percentile were associated with increased odds of any ASD diagnosis. Further, IL-1β (OR = 2.31, *p* = 0.017), IL-7 (OR = 2.28, *p* = 0.012), IL-10 (OR = 2.23, *p* = 0.018), IL-13 (OR = 2.42, *p* = 0.006), and MCP-1 (OR = 2.09, *p* = 0.041), were associated with increased odds of ASD with ID (Table 3). Finally, GMCSF (OR = 2.06, *p* = 0.041) and TNF-α (OR = 2.31, *p* = 0.014) were associated with ASD with ADHD (Table 3).

**Table 2:** Serum sample characteristics of mothers to individuals diagnosed with ASD and mothers to unaffected individuals in the study sample.

	**Unaffected** **(*n* = 429)**	**ASD** **(*n* = 318)**	***p*-value^a^**	**ASD only** **(*n* = 100)**	**ASD with ID** **(*n* = 101)**	**ASD with ADHD** **(*n* = 117)**	***p*-value^b^**
**Cytokines with <70% imputed values [median concentration (IQR)]**							
IL-1β (pg/ml)	2.3 (0.8, 5.8)	2.6 (1.0, 6.2)	0.27	2.5 (0.9, 5.9)	2.1 (1.2, 7.3)	2.9 (0.9, 6.8)	0.72
IL-2 (pg/ml)	5.4 (3.4, 9.9)	6.0 (3.6, 10.7)	0.20	6.1 (3.7, 10.3)	5.8 (3.8, 9.6)	6.6 (3.5, 11.3)	0.48
IL-6 (pg/ml)	5.0 (2.6, 14.1)	5.7 (2.6, 14.6)	0.54	4.7 (2.0, 10.4)	6.2 (2.6, 18.4)	6.0 (2.8, 17.9)	0.24
IL-7 (pg/ml)	7.3 (4.3, 11.6)	7.9 (4.5, 12.9)	0.15	8.4 (5.0, 13.4)	8.0 (4.3, 15.2)	7.9 (4.9, 12.3)	0.46
IL-8 (pg/ml)	222.9 (25.6, 1152.0)	164.1 (26.3, 917.3)	0.32	157.9 (33.6, 697.9)	240.8 (25.3, 1140.4)	121.9 (25.0, 556.6)	0.49
IL-17 (pg/ml)	18.0 (11.5, 26.9)	17.2 (11.2, 26.9)	0.96	19.8 (13.6, 29.4)	16.4 (11.2, 30.1)	15.8 (9.5, 25.4)	0.21
GCSF (pg/ml)	8.3 (4.8, 12.0)	8.7 (5.2, 12.8)	0.30	8.7 (5.3, 12.9)	7.8 (5.0, 12.4)	9.3 (6.3, 13.4)	0.37
GMCSF (pg/ml)	17.8 (6.0, 34.4)	16.6 (4.9, 39.9)	0.79	14.6 (3.5, 37.4)	22.4 (6.0, 43.0)	16.2 (6.3, 38.8)	0.26
MCP-1 (pg/ml)	53.7 (29.2, 91.5)	53.1 (30.1, 105.6)	0.71	51.7 (28.9, 88.8)	55.0 (32.8, 114.6)	55.4 (31.6, 100.1)	0.78
MIP-1β (pg/ml)	146.8 (103.9, 208.7)	145.8 (101.6, 202.8)	0.66	150.4 (97.0, 220.8)	140.8 (103.2, 201.1)	139.1 (101.6, 188.1)	0.73
**Cytokines with >70% imputed values [samples at or above the 90th percentile**, ***n*** **(%)]**							
IL-4	44 (10.3%)	39 (12.3%)	0.39	9 (9.0%)	14 (13.9%)	16 (13.7%)	0.51
IL-5	44 (10.3%)	35 (11.0%)	0.74	9 (9.0%)	16 (15.8%)	10 (8.5%)	0.29
IL-10	43 (10.0%)	47 (14.8%)	0.048	13 (13.0%)	18 (17.8%)	16 (13.7%)	0.16
IL-12	43 (10.0%)	46 (14.5%)	0.064	13 (13.0%)	14 (13.9%)	19 (16.2%)	0.26
IL-13	43 (10.0%)	45 (14.2%)	0.084	12 (12.0%)	22 (21.8%)	11 (9.4%)	0.009
IFN-γ	43 (10.0%)	39 (12.3%)	0.33	8 (8.0%)	12 (11.9%)	19 (16.2%)	0.19
TNF-α	43 (10.0%)	45 (14.2%)	0.084	11 (11.0%)	13 (12.9%)	21 (17.9%)	0.13

a *Pearson's chi-squared test was used for categorical variables, comparing the frequency distributions among unaffected individuals to the distributions among all ASD-affected individuals. Kruskal-Wallis tests were used to compare concentrations among unaffected individuals to concentrations among all ASD-unaffected individuals*.

b *Pearson's chi-squared test was used for categorical variables, comparing the frequency distributions among unaffected individuals to the distribution among the stratified ASD outcome groups. Kruskal-Wallis tests were used to compare concentrations among unaffected individuals to concentrations among the stratified ASD outcome groups*.

*ASD, autism spectrum disorders; ADHD, attention-deficit/hyperactivity disorder; ID, intellectual disability; IQR, interquartile range; IL-1β, Interleukin-1β; IL-2, Interleukin-2; IL-4, Interleukin-4; IL-5, Interleukin-5; IL-6, Interleukin-6; IL-7, Interleukin-7; IL-8, Interleukin-8; IL-10, Interleukin-10; IL-12, Interleukin-12; IL-13, Interleukin-13; IL-17, Interleukin-17; GCSF, Granulocyte Colony-Stimulating Factor; GMCSF, Granulocyte Monocyte Colony-Stimulating Factor; IFN-γ, Interferon-γ MCP-1, Monocyte Chemoattractant Protein 1; MIP-1β, Macrophage Inflammatory Protein 1β; TNF-α, Tumor Necrosis Factor-α*.

**Table 3:** The adjusted relationship between cytokines and odds of ASD, stratified by co-occurrence of ID and ADHD, when comparing mothers of 318 ASD-cases to mothers of 429 unaffected individuals selected from the cohort.

	**Any ASD** **[OR (LCI, UCI)]**	***p*-value**	**ASD only** **[OR (LCI, UCI)]**	***p*-value**	**ASD with ID** **[OR (LCI, UCI)]**	***p*-value**	**ASD with ADHD** **[OR (LCI, UCI)]**	***p*-value**
IL-1β	**1.71 (1.05, 2.77)**	0.030	1.51 (0.73, 3.14)	0.271	**2.31 (1.16, 4.60)**	**0.017**	1.53 (0.76, 3.05)	0.230
IL-2	1.23 (0.74, 2.04)	0.421	1.01 (0.46, 2.20)	0.986	1.09 (0.50, 2.39)	0.830	1.35 (0.67, 2.72)	0.408
IL-4	1.16 (0.70, 1.91)	0.571	0.86 (0.38, 1.92)	0.712	1.36 (0.67, 2.77)	0.396	1.09 (0.54, 2.23)	0.805
IL-5	1.04 (0.63, 1.74)	0.873	0.78 (0.35, 1.74)	0.547	1.87 (0.94, 3.69)	0.073	0.59 (0.26, 1.35)	0.212
IL-6	**1.68 (1.03, 2.75)**	**0.037**	1.32 (0.62, 2.83)	0.475	1.85 (0.92, 3.69)	0.082	1.77 (0.89, 3.52)	0.102
IL-7	1.23 (0.75, 2.02)	0.420	0.75 (0.34, 1.69)	0.490	**2.28 (1.20, 4.33)**	**0.012**	0.64 (0.28, 1.44)	0.281
IL-8	1.19 (0.69, 2.04)	0.533	0.89 (0.37, 2.18)	0.806	0.97 (0.40, 2.38)	0.955	1.69 (0.82, 3.47)	0.157
IL-10	**1.74 (1.08, 2.82)**	**0.024**	1.65 (0.80, 3.41)	0.175	**2.23 (1.14, 4.34)**	**0.018**	1.46 (0.72, 2.97)	0.297
IL-12	1.57 (0.97, 2.56)	0.068	1.84 (0.89, 3.80)	0.101	1.40 (0.68, 2.88)	0.354	1.64 (0.83, 3.24)	0.154
IL-13	1.39 (0.86, 2.26)	0.178	1.29 (0.62, 2.67)	0.502	**2.42 (1.29, 4.55)**	**0.006**	0.70 (0.31, 1.57)	0.383
IL-17	1.14 (0.68, 1.90)	0.626	1.31 (0.61, 2.81)	0.483	1.32 (0.64, 2.75)	0.453	0.86 (0.39, 1.92)	0.719
GCSF	1.11 (0.67, 1.84)	0.695	0.97 (0.44, 2.14)	0.947	0.96 (0.44, 2.10)	0.913	1.35 (0.66, 2.76)	0.405
GMCSF	1.39 (0.84, 2.30)	0.195	0.82 (0.35, 1.92)	0.647	1.62 (0.78, 3.37)	0.200	**2.06 (1.03, 4.11)**	**0.041**
IFN-γ	1.31 (0.79, 2.16)	0.291	0.84 (0.36, 1.96)	0.689	1.27 (0.61, 2.62)	0.520	1.37 (0.68, 2.76)	0.371
MCP-1	1.39 (0.83, 2.34)	0.208	0.78 (0.32, 1.90)	0.584	**2.09 (1.03, 4.24)**	**0.041**	1.40 (0.65, 3.00)	0.384
MIP-1β	1.21 (0.72, 2.05)	0.467	1.73 (0.83, 3.61)	0.146	0.80 (0.34, 1.87)	0.604	1.34 (0.63, 2.86)	0.448
TNF-α	**1.79 (1.1, 2.91)**	**0.020**	1.54 (0.71, 3.31)	0.271	1.87 (0.91, 3.85)	0.090	**2.31 (1.18, 4.50)**	**0.014**

*Dichotomous variables were created for each cytokine, using the distribution of z-scores among unaffected individuals to set the cut-offs, using values below the 90th percentile as the referent category. Models were adjusted for sex, maternal BMI, maternal psychiatric history, maternal region of origin, maternal age and family income quintile. p-values are shown for a Wald test with a null hypothesis that the cytokine categorical term was equal to zero, as a test of whether each cytokine was associated with the outcome. The bold values indicate the value of p < 0.05*.

*IL-1β, Interleukin-1β; IL-2, Interleukin-2; IL-4, Interleukin-4; IL-5, Interleukin-5; IL-6, Interleukin-6; IL-7, Interleukin-7; IL-8, Interleukin-8; IL-10, Interleukin-10; IL-12, Interleukin-12; IL-13, Interleukin-13; IL-17, Interleukin-17; GCSF, Granulocyte Colony-Stimulating Factor; GMCSF, Granulocyte Monocyte Colony-Stimulating Factor; IFN-γ, Interferon-γ MCP-1, Monocyte Chemoattractant Protein 1; MIP-1β, Macrophage Inflammatory Protein 1β; TNF-α, Tumor Necrosis Factor-α*.

In adjusted cubic spline models, only analytes with <70% imputed values were included (Supplementary Figures 7–10). A significant overall association (*p* = 0.032) was observed between IL-7 and ASD with ID (Supplementary Figure 9), with increased odds at the higher end of the distribution, consistent with the results in the categorical analysis. No other associations reached overall statistical significance, although some cytokines displayed similar patterns to those observed in the categorical analysis, with elevated odds of ASD at the high end of the distribution (MCP-1 and ASD with ID, Supplementary Figure 9; IL-6 and MCP-1 and ASD with ADHD, Supplementary Figure 10, Table 3).

Although several of the associations between cytokines and the outcomes were significant at *p* < 0.05 (Table 3), none survived the Bonferroni-adjusted significance thresholds based on either 17 or 68 statistical comparisons. Nor did any of the associations survive correction for multiple comparisons using the Benjamini-Hochberg procedure, with FDR specified at 5, 10 or 25%. Our lowest observed *p*-value (*p* = 0.006 for the association between IL-7 and ASD with ID in the categorical analysis) would only reach statistical significance (i.e., fall below the critical value) at an FDR of 41.7%.

### Principal Component Analysis

**Figure 2:**
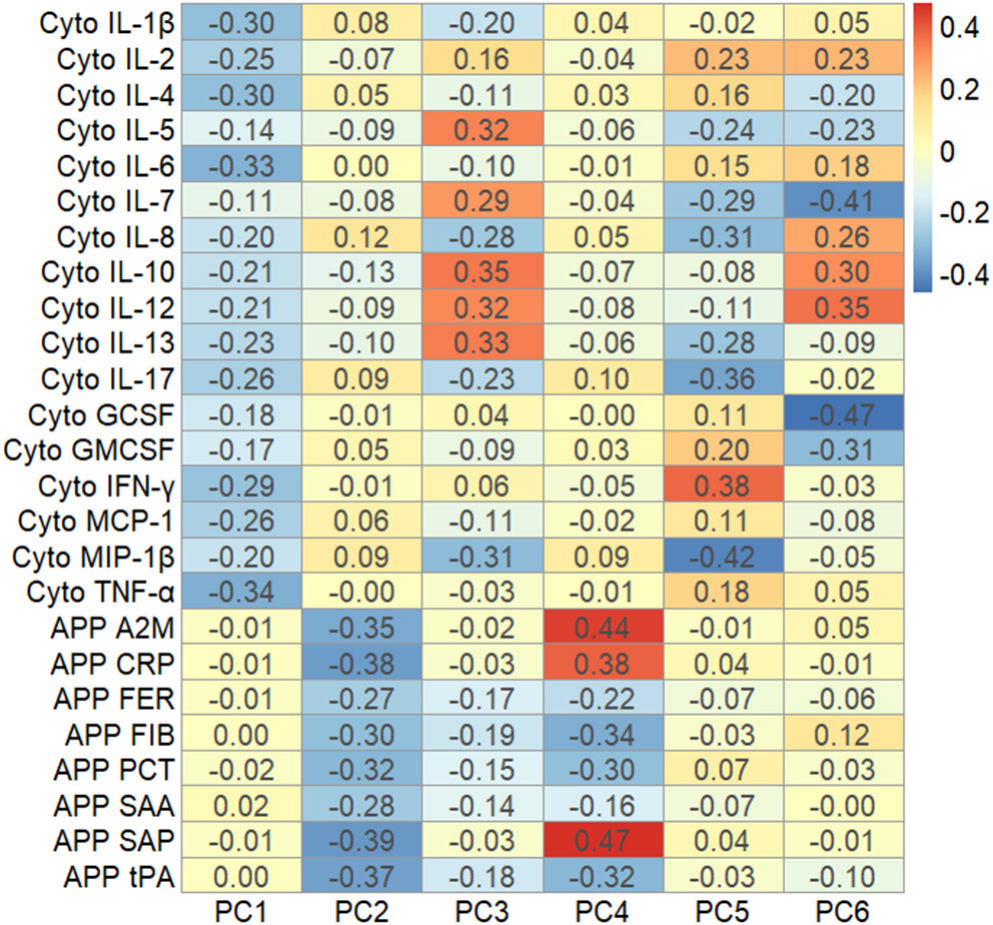
Heat map showing the factor loadings of the six largest principal components (PC1-PC6) derived from the variation in z-scores of all cytokines and acute phase proteins, using samples from mothers to cases and controls drawn in the first trimester of pregnancy (*n* = 747). A2M, α-2 Macroglobulin; CRP, C-Reactive Protein; FER, Ferritin; FIB, Fibrinogen; IL-1β, Interleukin-1β; IL-2, Interleukin-2; IL-4, Interleukin-4; IL-5, Interleukin-5; IL-6, Interleukin-6; IL-7, Interleukin-7; IL-8, Interleukin-8; IL-10, Interleukin-10; IL-12, Interleukin-12; IL-13, Interleukin-13; IL-17, Interleukin-17; GCSF, Granulocyte Colony-Stimulating Factor; GMCSF, Granulocyte Monocyte Colony-Stimulating Factor; IFN-γ, Interferon-γ MCP-1, Monocyte Chemoattractant Protein 1; MIP-1β, Macrophage Inflammatory Protein 1β; PCT, Procalcitonin; SAA, Serum Amyloid A; Serum Amyloid P; tPA, Tissue Plasminogen Activator; TNF-α, Tumor Necrosis Factor-α.

**Figure 3:**
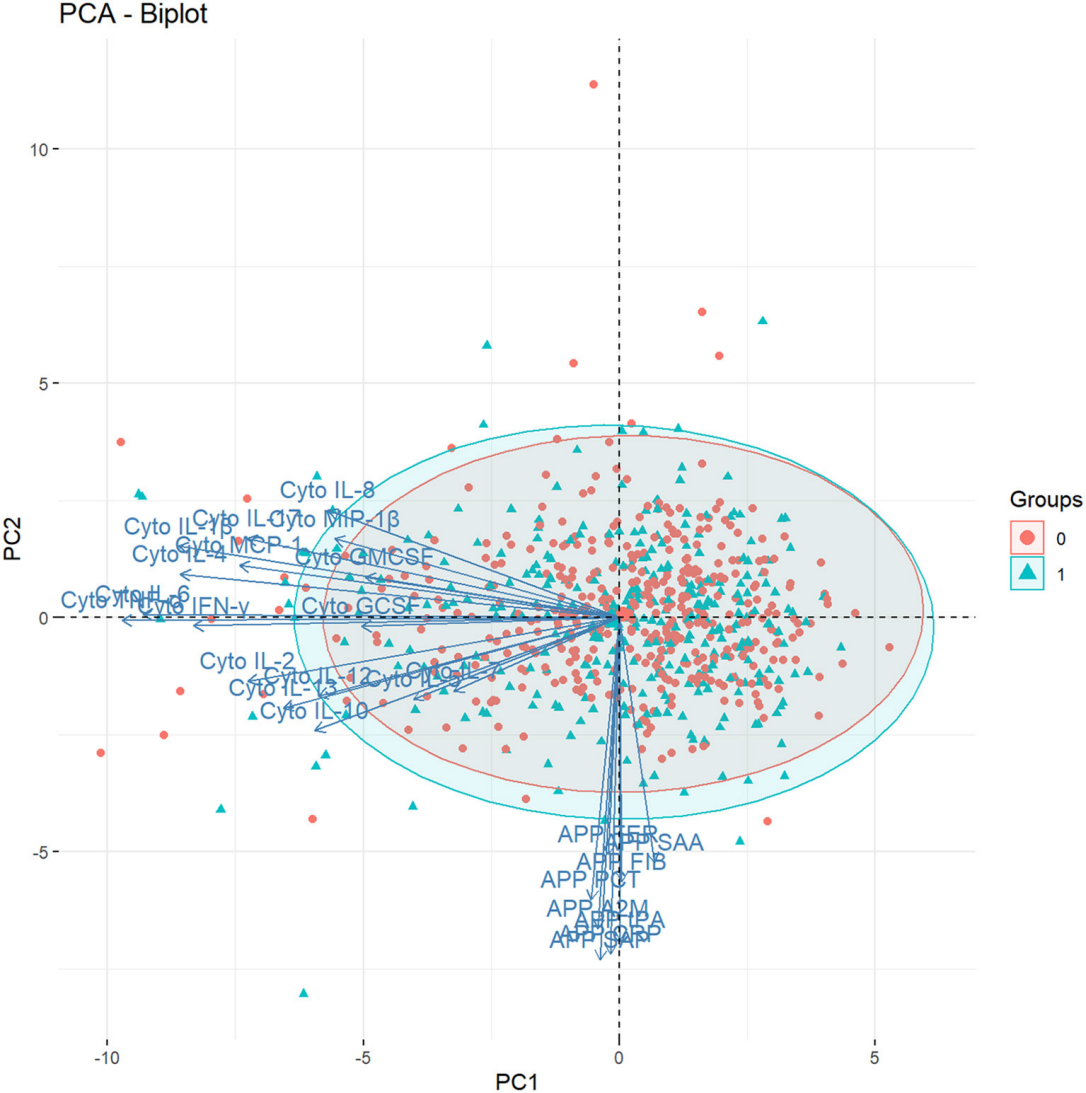
Biplot showing the factor loadings by the individual cytokines and acute phase proteins on the first two principal components (PC1 and PC2), and the values of these components for each individual in the study [*n* = 747, Groups: 0 = unaffected controls (red circles); 1 = ASD-cases (blue triangles)], in the two-dimensional component space generated by PC1 and PC2. A2M, α-2 Macroglobulin; APP, Acute Phase Protein; CRP, C-Reactive Protein; Cyto, Cytokine; FER, Ferritin; FIB, Fibrinogen; IL-1β, Interleukin-1β; IL-2, Interleukin-2; IL-4, Interleukin-4; IL-5, Interleukin-5; IL-6, Interleukin-6; IL-7, Interleukin-7; IL-8, Interleukin-8; IL-10, Interleukin-10; IL-12, Interleukin-12; IL-13, Interleukin-13; IL-17, Interleukin-17; GCSF, Granulocyte Colony-Stimulating Factor; GMCSF, Granulocyte Monocyte Colony-Stimulating Factor; IFN-γ, Interferon-γ MCP-1, Monocyte Chemoattractant Protein 1; MIP-1β, Macrophage Inflammatory Protein 1β; PCT, Procalcitonin; SAA, Serum Amyloid A; Serum Amyloid P; tPA, Tissue Plasminogen Activator; TNF-α, Tumor Necrosis Factor-α.

The first four principal components, each explaining >5% of the variation, together explained over 50% of the variation in cytokines and APPs (Supplementary Figure 11). PC1 explained about 24% of the variation in the immune markers (Supplementary Figure 12), and was dominated by variation in cytokines, with the largest factor loadings from IL-1β, IL-4, IL-6 and TNF-α (Figure 2). PC2 explained 10% of the variation (Supplementary Figure 12) and was mostly influenced by the APP, with the largest factor loadings from α-2-Macroglobulin (A2M), C-Reactive Protein (CRP), Serum Amyloid A (SAA) and Tissue Plasminogen Activator (tPA) (Figure 2). All factor loadings of the APP on PC2 were negative, indicating that an increase in APP's would lead to lower PC2 values. The cytokines and APP were completely separable when the factor loadings of each individual immune marker were plotted in the 2-dimensional component space generated by PC1 and PC2 (Figure 3). However, there was no separation by case-status when PC1 and PC2 were plotted in the same component space (Figure 3). Similarly, we observed no separation by case-status for the remaining combinations of the four largest components (PC1-PC4) (Supplementary Figures 13A–E).

**Figure 4:**
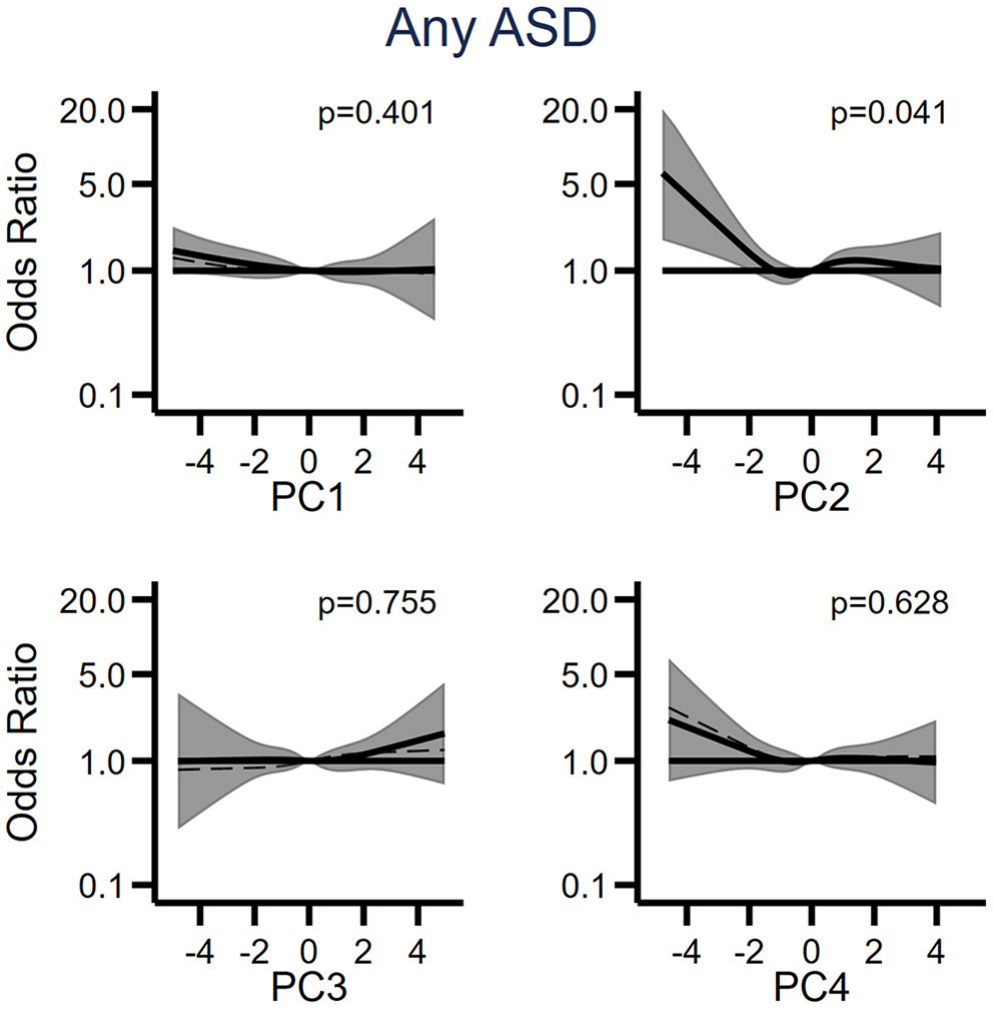
The relationship between the first four principal components (PC1-PC4) derived from cytokines and acute phase proteins, and odds of ASD when comparing 318 individuals affected by ASD to 429 unaffected individuals selected from the cohort. Each panel displays the odds of ASD according to principal component scores, flexibly fit using restricted cubic spline models with four knots and a score = 0 as the referent. The dashed line represents the unadjusted estimate of the relationship between each principal component and odds of ASD. The solid line represents the fully adjusted model, adjusted for sex of fetus; family income quintile; maternal BMI, psychiatric history, region of origin, and age. The gray bands represent the 95% confidence interval for the fully adjusted model. *P*-values are shown for a Wald test with a null hypothesis that all spline terms were jointly equal to zero, as a test of whether each principal component was generally associated with the outcome. PC, principal component.

We retained the first four components (PC1-PC4), which were approximately normally distributed (Supplementary Figure 14) and examined their potential relation with ASD using restricted cubic spline models to account for potential non-linear relationships (Figure 4; Supplementary Figures 15–17). We observed a significant (*p* = 0.041) association between low levels of PC2 and any diagnosis of ASD (Figure 4), and similar trends of increased odds of the stratified outcomes (ASD only, ASD with ID and ASD with ADHD) at low levels of PC2 (Supplementary Figures 15–17). Because the loadings of PC2 increased with decreasing APP levels, the overall associations between PC2 and odds of ASD outcomes indicate an increase in odds of ASD with generally increasing APP levels. We did not observe any relationships between any of the other components and the outcomes.

### Random Forest Prediction Models

**Figure 5:**
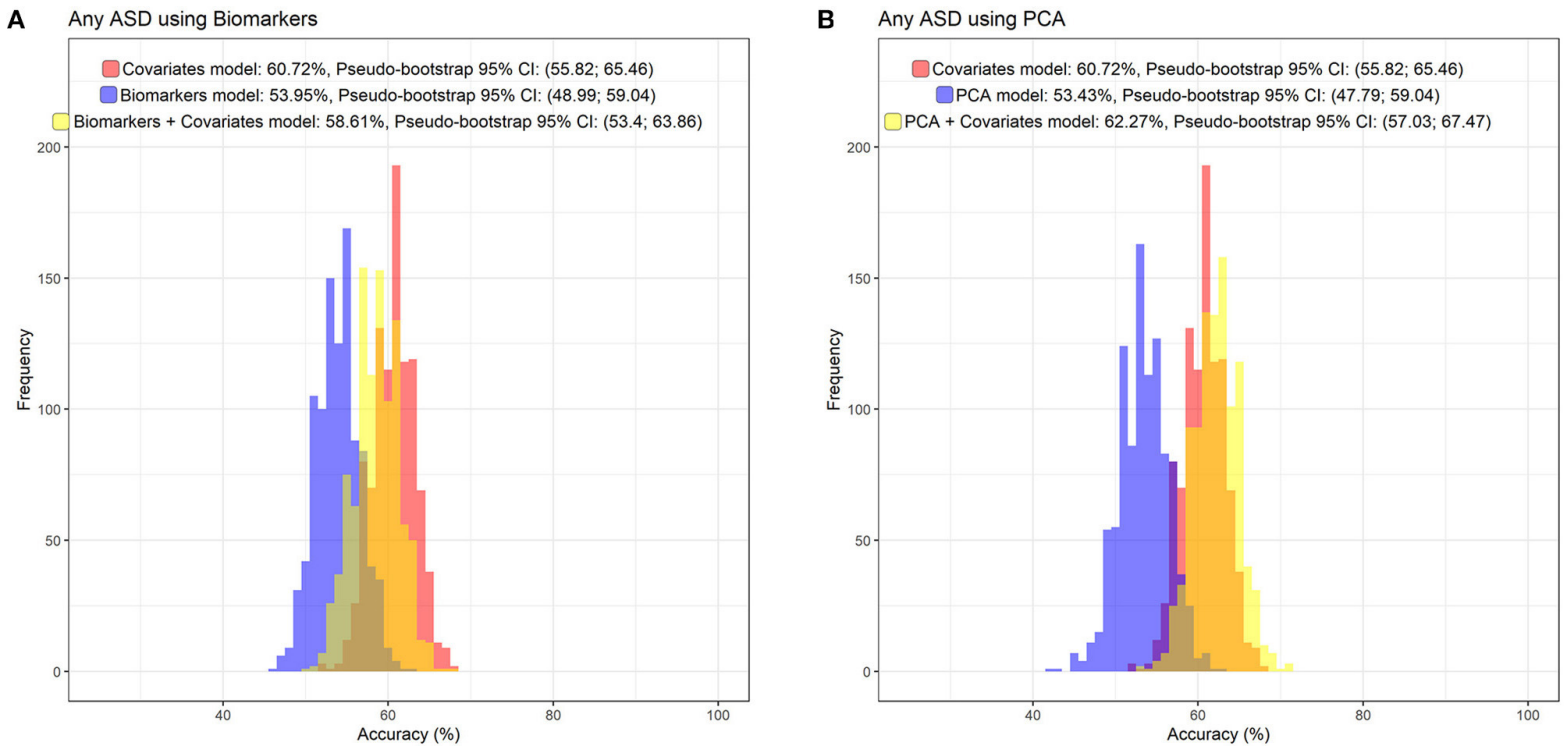
The distribution of prediction accuracies of Random Forest prediction models, including the non-biomarker (i.e., register) covariates used in the fully adjusted regression models (sex of fetus; family income quintile; maternal education, BMI, psychiatric history, region of origin, and age), and adding the original cytokine and APP z-score variables **(A)**, or the four largest principal components **(B)**.

**Figure 6:**
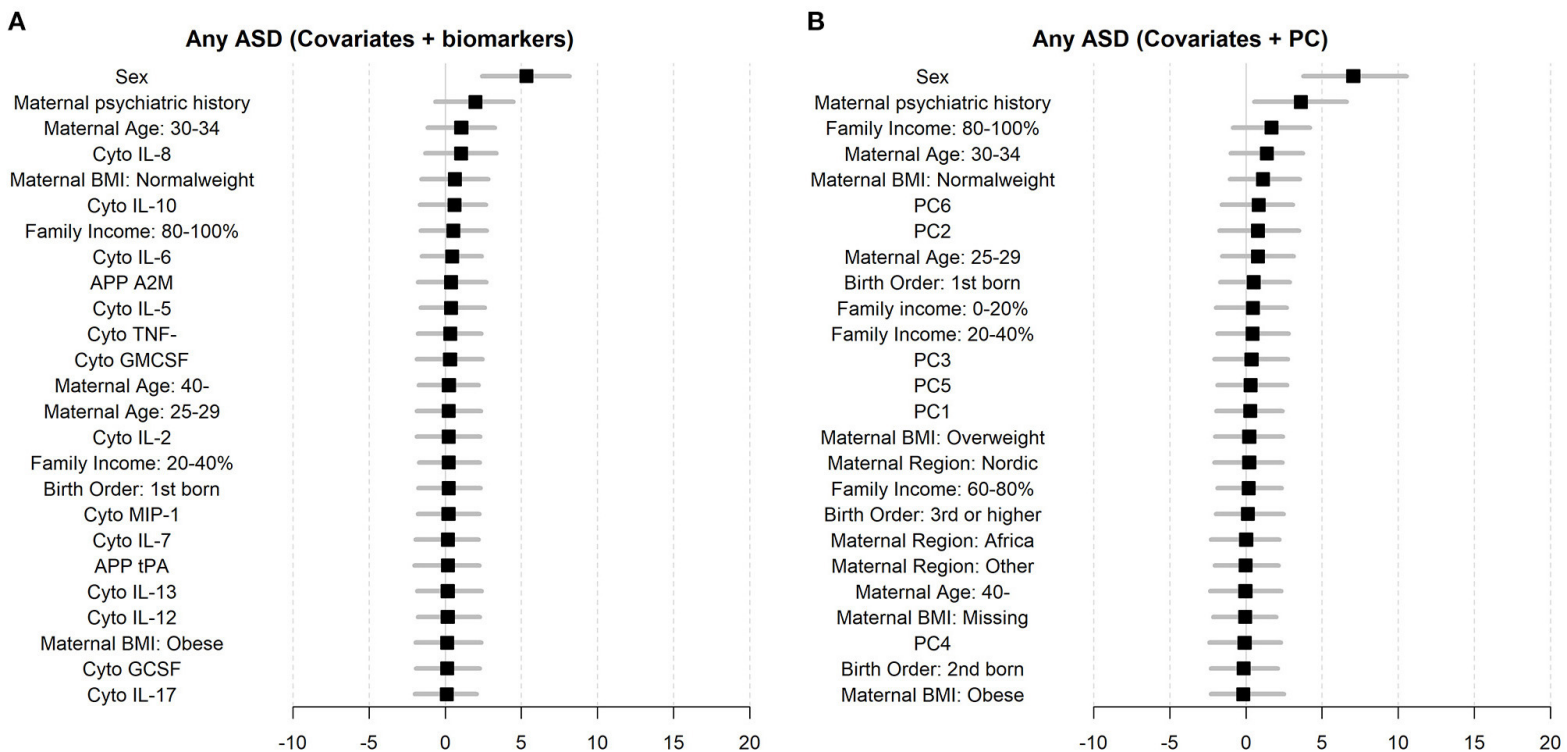
Variable importance measures for biomarkers and registered covariates **(A)**, and the six largest principal components (PC1–PC6) and registered covariates **(B)**.

The random forest prediction models (number of trees = 100) including only the register-based covariates (e.g., sex, birth order, income) had an overall prediction accuracy of ASD of 60.7% (Pseudo-bootstrap 95% CI 55.8; 65.5; Figure 5). There was no notable improvement in the accuracy of the prediction of ASD when either the immune markers collectively [mean accuracy 58.6% (Pseudo-bootstrap 95% CI 53.4; 63.9); Figure 5A], or the six largest principal components [mean accuracy 62.3% (Pseudo-bootstrap 95% CI 57.0; 67.5); Figure 5B], were added to the register-based covariates in the random forest prediction models. The average sensitivity of detecting ASD for the respective models were 41.8% (register-based covariates only), 42.6% (PC1–PC6 and register-based covariates), and 33.6% (biomarkers and register-based covariates). The average specificity of each model was 74.9% (covariates only) 77.1% (PC1–PC6 and register-based covariates), and 77.4% (individual biomarkers and register-based covariates). The variable importance measurements showed that sex of the child was the most important predictor for ASD, followed by maternal psychiatric history (Figures 6A,B). The algorithm also deemed a high family income at birth, maternal age and BMI to be among the top predictors of ASD in this sample.

### Sensitivity Analysis

When samples from all trimesters were included (*n* = 979), the observed associations were generally consistent with the main categorical analyses (restricted to the first trimester) regarding direction and magnitude, although some differences were observed (Supplementary Table 4). The associations between IL-7 or MCP-1 and ASD with ID, and the association between GMCSF and ASD with ADHD were no longer significant (at *p* = 0.05), whereas the associations between IL-12 and any ASD diagnosis, and GMCSF and ASD with ID, fell below the threshold for statistical significance (α = 0.05, Supplementary Table 4). As in the main categorical analysis, none of the associations survived correction for multiple comparisons.

When using samples from all trimesters, the results of the PCA were similar to the main analysis, though the associations with ASD outcomes observed at low levels of PC2 were less prominent (Supplementary Figure 18).

## Discussion

In this study, we measured 17 cytokines in first trimester maternal serum samples from 318 mothers to ASD-cases and 429 mothers to unaffected controls. After adjusting for a range of potential confounding factors, elevated levels of the pro-inflammatory cytokines IL-1β, IL-6, and TNF-α and the inhibitory cytokine IL-10 were associated with diagnosis of ASD. The results varied across diagnostic sub-groups, based on the presence of comorbid ID or ADHD. While no significant associations between cytokines and ASD without ID or ADHD were observed, elevated levels of the pro-inflammatory cytokines IL-1β and MCP-1, the hematopoetic growth factor IL-7, the inhibitory IL-10, and the Th2-cytokine IL-13, were associated with ASD with co-occurring ID. Elevated levels of the monocyte growth factor GMCSF and the pro-inflammatory TNF-α were associated with ASD with co-occurring ADHD. Though several associations between individual cytokines and ASD were observed, there was no convincing general pattern of association with ASD after accounting for multiple comparisons. In PCA analysis, we observed separation of cytokines from additional immune markers measured in this cohort (APP), though no convincing separation of cases and controls. Only one extracted principal component, whose loading was dominated by APP, was associated with ASD. Using the immune markers collectively did not improve the prediction of ASD in our sample, beyond that observed for models including only maternal and child characteristics obtained from register data.

### Comparison With Previous Studies

**Figure 7:**
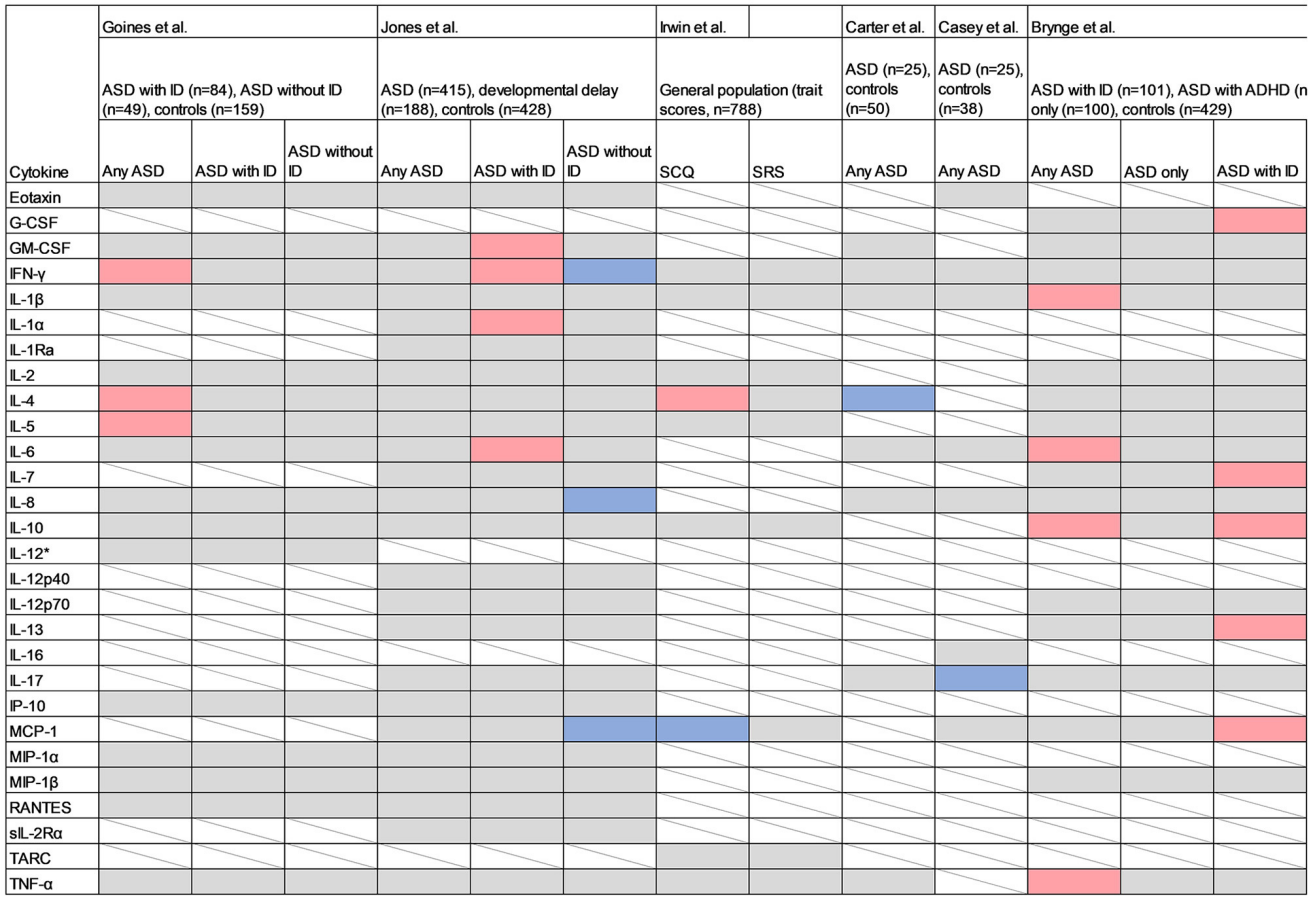
Studies measuring cytokines in maternal serum samples. Red cell color indicates a positive association (*p* < 0.05) between the cytokine and the outcome, blue indicates an inverse association, grey indicates no association (*p* > 0.05), and white with strikethrough indicates that the cytokine was not measured. SCQ, Social Communication Questionnaire; SRS, Social Responsiveness Scale; IL-1α, Interleukin 1α; IL-1β, Interleukin 1β; IL-2, Interleukin 2; IL-4, Interleukin 4; IL-6, Interleukin 6; IL-7, Interleukin 7; IL-8, Interleukin 8; IL-10, Interleukin 10; IL-12p40, Interleukin 12p40; IL-12p70, Interleukin 12p70; IL-13, Interleukin 13; IL-16, Interleukin 16; IL-17, Interleukin-17; IL-17A, Interleukin-17A; GCSF, Granulocyte Colony-Stimulating Factor; GMCSF, Granulocyte Monocyte Colony-Stimulating Factor; IFN-γ, Interferon-γ; IP-10, Interferon gamma-induced protein 10; MCP-1, Monocyte Chemoattractant Protein 1; MIP-1α, Macrophage Inflammatory Protein 1α; MIP-1β, Macrophage Inflammatory Protein 1β; RANTES, Regulated on Activation, Normal T-cell Expressed and Secreted; sIL-2Rα, Soluble Interleukin 2 Receptor-Alpha; TARC, Thymus and Activation Regulated Chemokine; TNF-α, Tumor Necrosis Factor α.

The previous studies investigating cytokines in maternal serum samples used samples collected later in pregnancy (2nd and 3rd trimesters) compared to the present study (Figure 7). One of the studies used a quantitative scale for measuring autistic traits in a general population sample, whereas the others used clinically evaluated neurodevelopmental diagnoses. Goines et al. analyzed 17 cytokines (using a different assay compared to the present study) in archived maternal serum samples, collected at gestational weeks 15–19, from mothers to cases with ASD (*n* = 84), ID without ASD (*n* = 49), and population controls (*n* = 159) (23). IFN-γ, IL-4 and IL-5 were significantly associated with any diagnosis of ASD, independent of early/late onset of ASD or presence of ID. In 2016, the same research group performed a follow-up study, analyzing 22 cytokines from mothers to ASD-cases (*n* = 415), developmental delay (*n* = 188) and controls (*n* = 428) (24). No significant associations were found for any of the cytokines and any ASD-diagnosis. When the sample was stratified based on co-morbidity, GMCSF, IL-1α, IL-6 and IFN-γ were significantly associated with increased odds of ASD with ID, and IFN-γ, IL-8 and MCP-1 were associated with decreased odds of ASD without ID. Some of the associated cytokines overlapped with those observed in the present study (Figure 7), but the results are inconsistent when considering the specific diagnostic sub-groups. For example, IL-6 was associated with an overall diagnosis of ASD in the present study, but only with a diagnosis of ASD with ID in the study by Jones et al.

In the “Seychelles Child Development Study Nutrition Cohort 2”, Irwin et al. measured 13 inflammatory markers including 10 cytokines in a cohort of 788 mother-child pairs, at a mean of 28 weeks of gestation, and related these to the trait scales Social Communication Questionnaire (SCQ) and Social Responsiveness Scale (SRS), at age 7 (25). Both the exposures and outcomes were considered as continuous linear variables. A significant association was reported for IL-4 and autistic traits as measured by SCQ, which is in line with the findings by Goines et al. However, an inverse association between MCP-1 and SCQ contradicts the positive association between MCP-1 and ASD without ID reported by Jones et al. Because Irwin et al. evaluated autistic traits in the general population, any direct comparison with studies employing a case-control design are difficult.

In a recent case-control study, Carter et al. (26) investigated the association between eight maternal cytokines measured at 20 weeks of gestation in 25 mothers to ASD-cases and 50 mothers to controls, matched by infant sex, gestational age, birthweight and maternal BMI. The median cytokine concentrations were compared between groups using non-parametric tests. Decreased levels of mid-gestational IL-4 were associated with ASD, which contradicts the findings by Goines et al. and Irwin et al. (23, 25). Casey et al. combined maternal serum samples from two mother-child cohorts including 25 mothers to ASD-cases and 38 mothers to controls, and investigated associations between eight maternal cytokines at 15 and 20 weeks of gestation and odds of ASD in offspring (47). Decreased levels of IL-17A at 20 weeks of gestation were associated with increased odds of ASD.

This is the first study to date to investigate maternal serum cytokines during the first trimester of pregnancy and risk of ASD in the offspring. In the present study, we analyzed cytokines in a study sample nested within a large population-based cohort. None of the significant associations in the present study overlapped with those reported previously. There are methodological differences between the studies that may explain these discrepancies. As in previous case-control studies, our archived serum samples were collected as part of an antenatal screening program, and handling of samples before final storage at −80°C cannot be accounted for. This might have influenced the reliability of the measurements (48). Finally, multiple exposures and endpoints are assessed, and none of the previous studies correct for this. Hence, there is a possibility that the positive associations reported previously represent chance findings due to heterogeneity in inclusion criteria and sampling variability.

### Interpretation

In this cohort of pregnant women, we measured a wide range of cytokines, including mediators of both innate and adaptive immune pathways. We observe several associations between individual immune markers and ASD. However, the associations were weak to moderate in general, and none survived correction for multiple comparisons. This is in line with previous studies on maternal cytokines and ASD, although none of the previous studies corrected for multiple comparisons. Overall, we found no strong evidence for maternal immune activation among mothers to individuals with ASD in this cohort, although there was a tendency for odds ratios estimates to be above one, similar to other studies (23, 24).

Although we observe increased levels of several of the pro-inflammatory cytokines and ASD, the specific results differ considerably according to diagnostic sub-group. Indeed, recent data suggests that ASD without ID is more familial and may have a different genetic architecture than ASD with ID (4, 49, 50). The findings may indicate the presence of a first-trimester gestational immune stimulus among some mothers to ASD-cases. This observation might reflect the presence of an (unmeasured) external or inherent “driver” of cytokine levels in these mothers, such as infection or genetic variants.

If any of the cytokines are causally related to ASD, the mechanism by which they can potentially affect children's neurodevelopment remains to be established. In experimental animals, IL-6 can cross the placental barrier and influence fetal brain development potentially through transmission of an inflammatory signal to the fetal compartment (21, 51). Maternal cytokines can also affect the cytokine production by decidual cells of the placenta, with potential consequences for the fetal levels. Several of the cytokines play important roles in key neurodevelopmental processes, e.g., neuronal migration and differentiation and synaptic pruning, and a shift in cytokine levels might interfere with such processes.

The overall picture indicates that instead of relying on individual markers, some general aspect of the maternal immune function and its interaction with the developing fetus may be relevant for ASD. To integrate the biological information from all our measured immune markers in one model, we employed an approach using PCA, also including measurements of maternal APP measured in the same cohort of women. A similar approach has been used in a few previous studies of cytokines in archived maternal or neonatal samples. Jones et al. used separate PCA for each diagnostic sub-group (ASD with ID, ASD without ID, developmental delay, controls) to investigate the presence of specific aggregated patterns of maternal cytokines. The authors concluded that there was no obvious specific cytokine-profile for any of the stratified groups. Krakowiak et al. conducted a PCA of 14 cytokines, though these were measured in neonatal samples from ASD-affected and unaffected children. Overall, there were no specific clustering patterns across diagnostic sub-groups (mild/moderate ASD, severe ASD, developmental delay, controls). Finally, Heuer et al. used partial least squares discriminant analysis (PLS-DA) to investigate if neonatal cytokine levels separated diagnostic groups (ASD cases, controls). The authors concluded that there was no outcome-specific profile based on a plot of cases and controls in component space, but a variable importance analysis suggested that the cytokines IL-6 and IL-8 were the most important cytokines, which corresponded to their logistic regression analyses. In our study, maternal levels both IL-6 and IL-8 were among the most important predictors in our random forest models, although the confidence intervals overlapped with zero.

We observed a non-linear relationship between one of the extracted principal components, PC2, and odds of ASD, with increased odds of ASD associated with low PC2 scores. Low PC2 scores correspond to high levels of several of the APP, given the direction of the loading of the APPs on the second principal component, indicating that children's odds of ASD generally increase with increasing levels of maternal APP. The remaining components, including the cytokine-dominated PC1, showed no significant associations with ASD-case status, and cases and controls were not otherwise separated based on the first four components. This indicates that maternal APP levels may contain more information relevant for the children's risk of ASD than maternal cytokine levels. This may relate to their longer half-lives, higher base-line concentrations, and effector functions within the maternal innate immune system (52).

Finally, adding the immune markers or their derived principal components to other registered covariates regarding maternal and child characteristics did not improve the prediction of ASD-case status in Random Forest prediction models. This indicates that knowledge regarding maternal immune biomarkers (including cytokines and APP) are unlikely to be informative in terms of early detection of ASD. However, the weak predictive capability does not rule out a role for maternal immune status in the etiology of ASD. The potential dysregulation of immune processes may be more subtle than what can be detected in our current sample, and the maternal serum samples from early pregnancy may not reflect the conditions in the developing fetal brain. There is a possibility that each of the markers has a very small effect size that we are unable to capture given our restricted sample size, and that each of the small effects add up to a cumulative effect that is only detectable in a substantially larger population, analogous to single nucleotide polymorphisms in genome wide association studies.

### Strengths and Limitations

We use a validated case-finding procedure within a healthcare system with universal coverage and regular developmental screening. This increases the likelihood of identifying ASD cases in the population. Using our large, well-characterized population-based cohort, we were able to assess confounding by a range of different environmental factors and investigate how they influence the serum levels of cytokines. By analyzing multiple immune markers, we increased the likelihood of detecting a signal of activation of the maternal immune system. On the other hand, by doing multiple statistical comparisons, we also increased the probability of chance findings. None of the associations survived correction for multiple comparisons, either using a traditional (Bonferroni) or a less conservative (FDR) approach. A strength of our study is the PCA and Random Forest analyses, which move beyond the conventional one-by-one analyses and allows for an integrated interpretation of the immune markers.

We could adjust for a wide range of potential confounders, such as maternal BMI and fetal sex, with documented associations with both the maternal immune status and autism (8, 30, 53). However, there is a possibility for residual confounding by unmeasured genetic or environmental factors. We do not have serum samples available from multiple pregnancies and can therefore not perform sibling-comparisons. Our previous work in NDBS suggests that this would likely be valuable in order to address issues of confounding by shared familial factors (32). Indeed, recent research suggests that adverse prenatal exposures are associated with maternal genetic liability for neurodevelopmental disorders (54), further stressing the importance of adjustment for genetic factors in studies of maternal immune markers and children's risk of ASD. Despite our large overall sample, the number of individuals in the stratified groups is limited, and we may be underpowered to detect more subtle relationships, particularly in the diagnostic sub-groups.

By restricting the analysis to the first trimester, we reduced heterogeneity and variation in cytokines related to the progression of pregnancy. On the other hand, the strategy also reduced the sample size and statistical power and prevented detection of differences during later stages of pregnancy. The majority of serum samples (76.3%) in this study were drawn in the first trimester. The observed associations between several of the maternal cytokines and gestational age, as well as other covariates, stress the importance of taking key covariates into account in any assessment. Including samples from all trimesters in a sensitivity analysis yielded results similar to the main analysis in terms of the direction and magnitude of the estimated odds ratios, though some relationships were less apparent when considering the full cohort and a few became more apparent.

Cytokines are powerful regulators of the immune system and generally have short half-lives and low baseline levels at homeostatic conditions. Since cytokines are often involved in local (paracrine) cell-signaling pathways, systemic levels may not necessarily reflect concentrations at peripheral sites of inflammation, or in the embryo and the fetal/placental unit. Thus, measuring concentrations in blood/serum samples can be both technically challenging and difficult to interpret. Overall, there was a large proportion of non-detectable values for many of the cytokines. The inter-assay coefficient of variation was markedly higher for IFN-γ compared to the other cytokines, and the results for IFN-γ must therefore be interpreted with some caution.

Moreover, because these samples were collected as part of a clinical screening program, storage procedures may have varied. Samples could be stored at room temperature or in a refrigerator up to several days before freezing at −80°C, though this procedure was not within our control, and we do not have information on the handling of individual samples prior to their arrival in our laboratory. Since cytokines are vulnerable to degradation at ambient temperatures, there is a possibility that the levels measured in the samples do not reflect the actual levels at the time of sampling/venipuncture. The degradation of cytokines in the samples due to these issues would attenuate any real associations toward the null, and further reduce our ability to detect case/control differences.

## Conclusions

While we observed a number of individual associations between maternal cytokines measured in early pregnancy and children's risk of ASD, none survived corrections for multiple comparisons. Considering the individual associations, our results do not provide strong support for the maternal immune activation hypothesis in ASD, especially when compared to the often divergent results of previous studies. The relationships we observed varied with the presence or absence of co-occurring neurodevelopmental diagnoses, including both ADHD and ID. We also observed variation in the maternal cytokine levels with key covariates, such as maternal characteristics and the gestational week at serum sampling. This emphasizes the importance of considering both potential genetic and environmental influences on the maternal immune system when attempting to interpret associations between maternal immune biomarkers and children's risk of ASD in future studies.

When taking a more integrated view of biomarkers reflecting the maternal immune response in the first trimester, we observed that maternal cytokines as a class were not strongly associated with children's risk of ASD, though higher levels of another class of immune biomarkers, the acute phase proteins, were associated with children's risk for ASD. Using all of these markers together did not markedly improve prediction models for ASD, indicating a limited utility for maternal immune biomarkers in early detection strategies for ASD given the limitations of studies to date in terms of sample size and the relatively small effect sizes for the individual markers.

## Data Availability Statement

The Swedish health and population register data used in this study are available from Statistics Sweden and the Swedish National Board of Health and Welfare. The authors are not allowed to distribute the data according to the ethical approval for this study and the agreements with Statistics Sweden and the Swedish National Board of Health and Welfare.

## Ethics Statement

The studies involving human participants were reviewed and approved by Stockholm Regional Review Board. Written informed consent for participation was not required for this study in accordance with the national legislation and the institutional requirements.

## Author Contributions

MB contributed to data acquisition, data analysis, and drafted the first manuscript. HK and RG contributed to study design, data acquisition, data analysis, and manuscript preparation. CD contributed to data acquisition, study design, and manuscript preparation. HS contributed to data acquisition, data analysis, and manuscript preparation. BL contributed to study design and manuscript preparation. All authors contributed to the article and approved the submitted version.

## Funding

This work was supported by grants from the Swedish Research Council [Grant Nos. 2016-01477, 2012-2264, and 523-2010-1052 (to CD), Grant Number 2017-02900 (to RG)], Autism Speaks [Basic and Clinical Grant No. 7618 (to BL)], and the Stanley Medical Research Institute (to HK). The funders had no role in the design and conduct of the study; collection, management, analysis, and interpretation of the data; preparation, review, and approval of the manuscript; or decision to submit the manuscript for publication.

## Conflict of Interest

The authors declare that the research was conducted in the absence of any commercial or financial relationships that could be construed as a potential conflict of interest.

## Publisher's Note

All claims expressed in this article are solely those of the authors and do not necessarily represent those of their affiliated organizations, or those of the publisher, the editors and the reviewers. Any product that may be evaluated in this article, or claim that may be made by its manufacturer, is not guaranteed or endorsed by the publisher.
