# Supplementary material for: Maternal Levels of Cytokines in Early Pregnancy and Risk of Autism Spectrum Disorders in Offspring
Source: Front Public Health. 2022 May 31;10:917563. doi: 10.3389/fpubh.2022.917563 (PMC9197505; doi:10.3389/fpubh.2022.917563)
Supplement: Supplementary file 1 [file Data_Sheet_1.pdf]

Supplementary Material

1 Supplementary Figures and Tables

1.1 Supplementary Figures

Supplementary Figure 1. Levels of one cytokine (MIP-1β) according to analytic plate. (**A**) Concentrations (pg/ml). (**B**) Log-2-values. (**C**) Plate-specific z-scores were calculated to allow comparisons across plates.

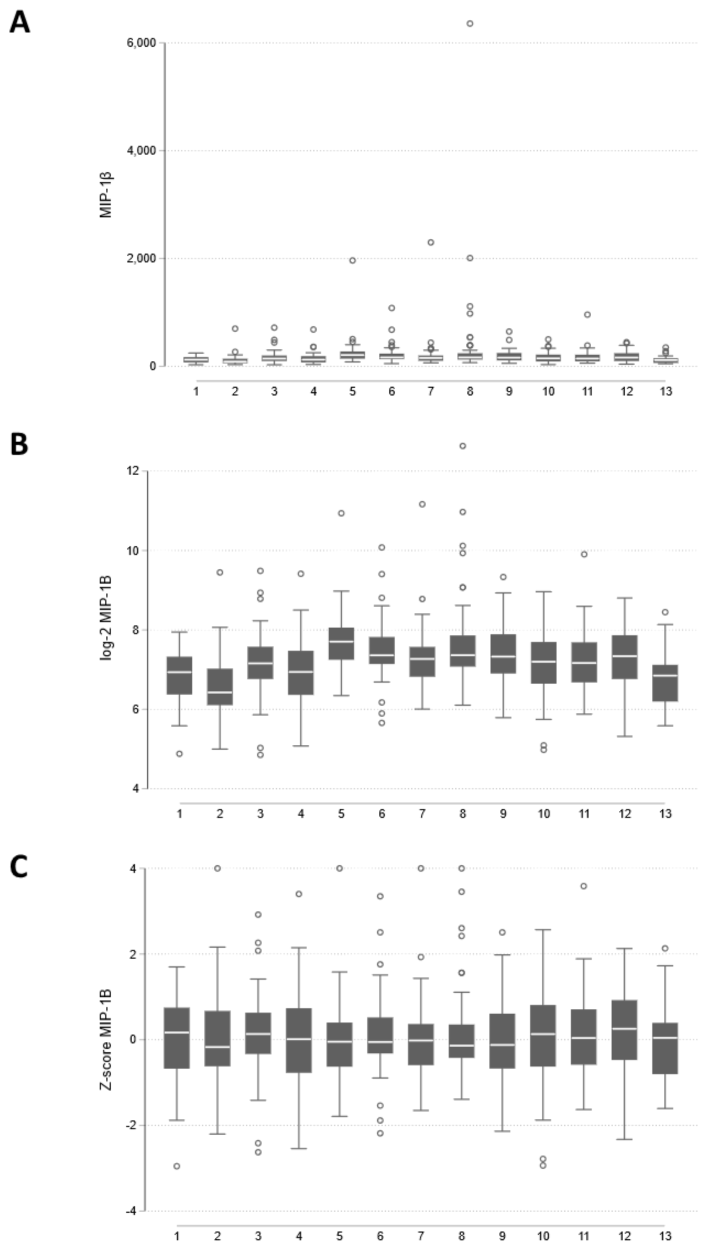

Supplementary Figure 2. The Spearman rho correlations of cytokines with each other, as measured in maternal serum samples of 429 unaffected controls. All measured values (**A**) and excluding values that were outside the limits of quantitation (**B**). Abbreviations: **IL-1β**: Interleukin-1β; **IL-2**: Interleukin-2; **IL-4**: Interleukin-4; **IL-5**: Interleukin-5; **IL-6**: Interleukin-6; **IL-7**: Interleukin-7; **IL-8**: Interleukin-8; **IL-10**: Interleukin-10; **IL-12**: Interleukin-12; **IL-13**: Interleukin-13; **IL-17**: Interleukin-17; **GCSF**: Granulocyte Colony-Stimulating Factor; **GMCSF**: Granulocyte Monocyte Colony-Stimulating Factor; **INF-γ**: Interferon-γ **MCP-1**: Monocyte Chemoattractant Protein 1; **MIP-1β**: Macrophage Inflammatory Protein 1β; **TNF-α**: Tumor Necrosis Factor-α.

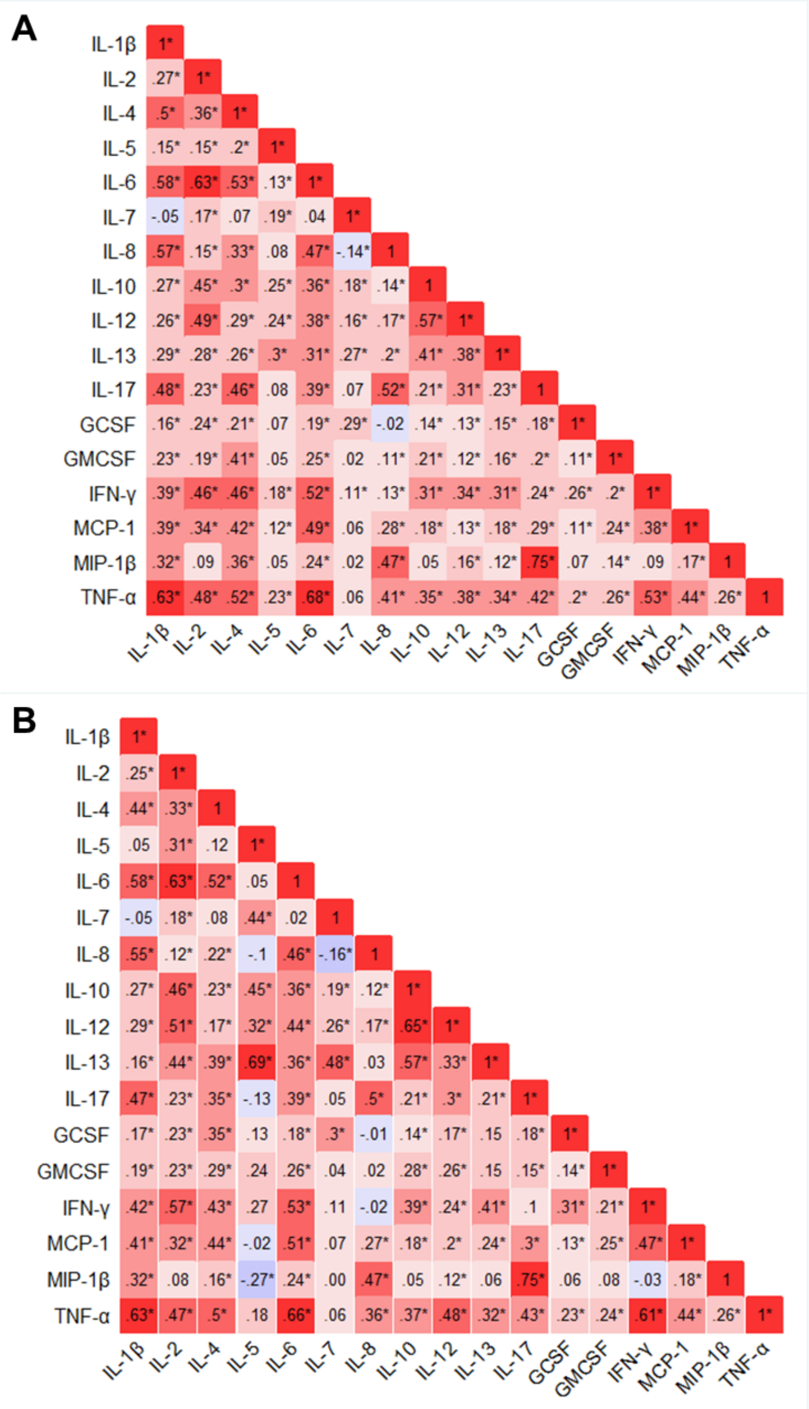

Supplementary Figure 3. Heat map showing the mean z-score of cytokines with <70% imputed values, by categories of the covariates, among mothers to 318 ASD-affected individuals in the cohort. Solid boxes indicate that the cytokine is associated with the covariate at p<0.05. Dashed boxes indicate that the cytokine is associated with the covariate at p<0.20. Abbreviations: **IL-1β**: Interleukin-1β; **IL-2**: Interleukin-2; **IL-6**: Interleukin-6; **IL-7**: Interleukin-7; **IL-8**: Interleukin-8; **IL-17**: Interleukin-17; **GCSF**: Granulocyte Colony-Stimulating Factor; **GMCSF**: Granulocyte Monocyte Colony-Stimulating Factor; **MCP-1**: Monocyte Chemoattractant Protein 1; **MIP-1β**: Macrophage Inflammatory Protein 1β.

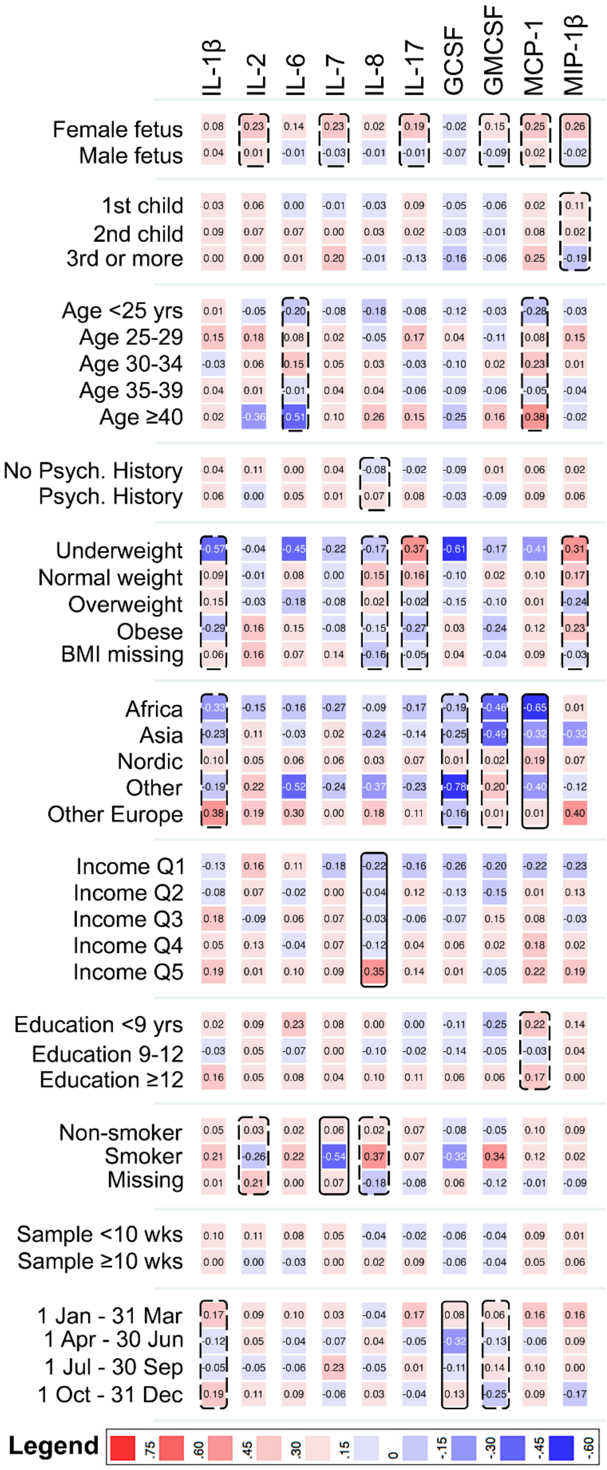

Supplementary Figure 4. Bar chart showing the proportion of samples at or above the 90th percentile for maternal cytokines with >70% imputed values, by categories of covariates among 429 ASD-unaffected controls. Abbreviations: **IL-4**: Interleukin-4; **IL-5**: Interleukin-5; **IL-10**: Interleukin-10; **IL-12**: Interleukin-12; **IL-13**: Interleukin-13; **INF-γ**: Interferon-γ; **TNF-α**: Tumor Necrosis Factor-α.

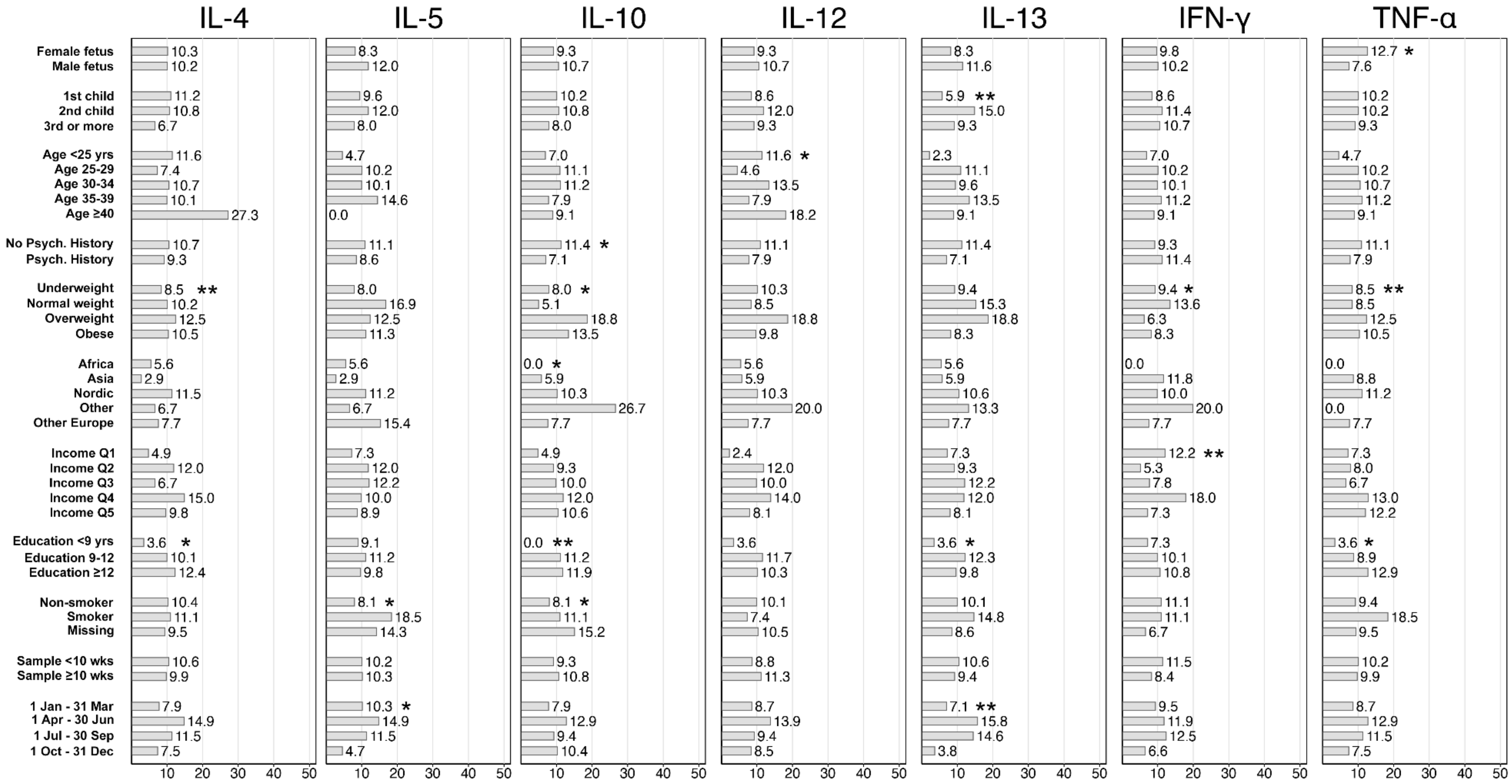

***** $p < 0.20$
** $p < 0.05$

Supplementary Figure 5. Proportion of samples at or above the 90th percentile for cytokines with >70% imputed values, by categories of covariates among 318 ASD-cases. . Abbreviations: **IL-4**: Interleukin-4; **IL-5**: Interleukin-5; **IL-10**: Interleukin-10; **IL-12**: Interleukin-12; **IL-13**: Interleukin-13; **INF-γ**: Interferon-γ; **TNF-α**: Tumor Necrosis Factor-α.

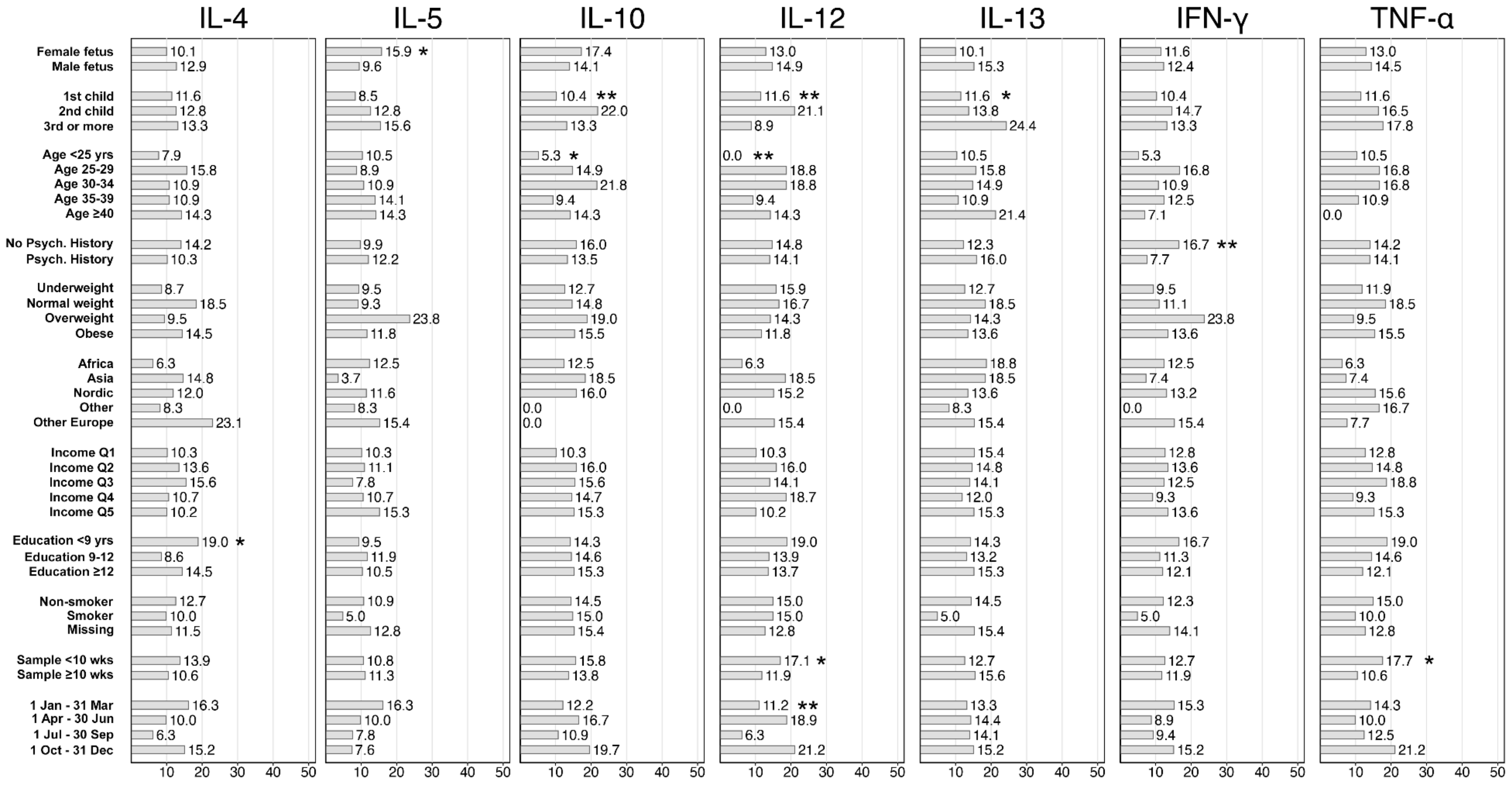

* $p < 0.20$
** $p < 0.05$

Supplementary Figure 6. The distribution of cytokines with <70% imputed values, measured in maternal serum samples, comparing ASD-unaffected individuals (0) to ASD-affected individuals (1).

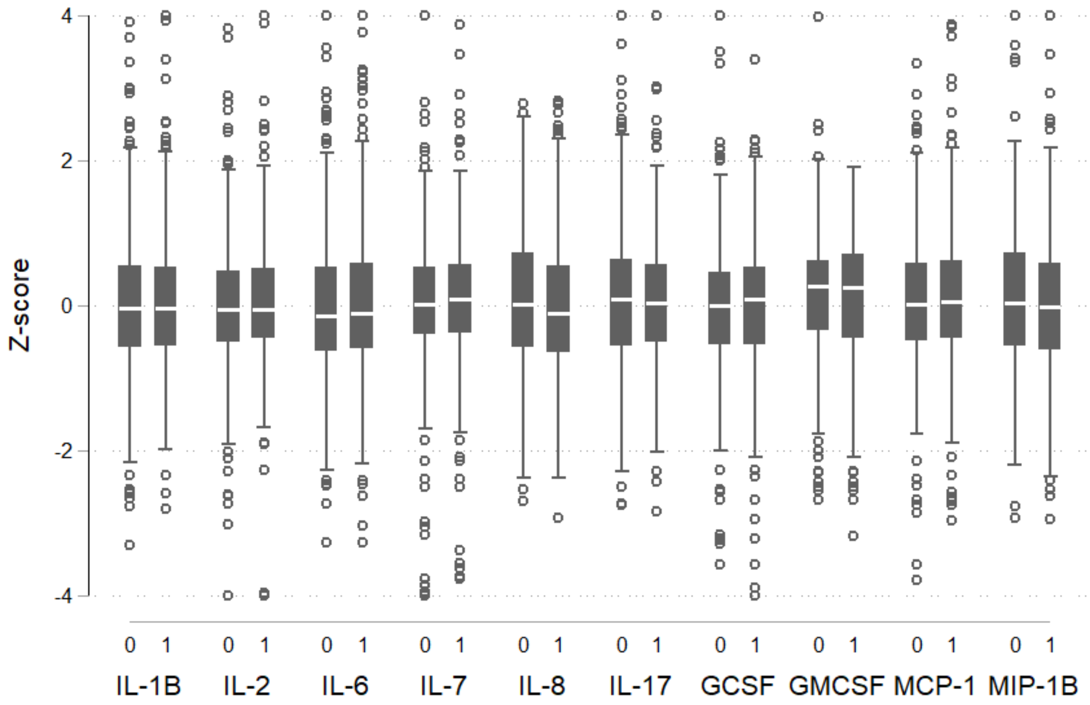

Supplementary Figure 7. The relationship between maternal cytokines and odds of ASD when comparing 318 individuals affected by ASD to 429 unaffected individuals selected from the cohort. Each panel displays the odds of ASD according to cytokine z-scores, flexibly fit using restricted cubic spline models with three knots and a z-score=0 as the referent. The dashed line represents the unadjusted estimate of the relationship between each cytokine and odds of ASD. The solid line represents the fully adjusted model, adjusted for sex of fetus; family income quintile; maternal BMI, psychiatric history, region of origin, and age. The gray bands represent the 95% confidence interval for the fully adjusted model. P-values are shown for a Wald test with a null hypothesis that all cytokine spline terms were jointly equal to zero, as a test of whether each cytokine was generally associated with the outcome. Abbreviations: **IL-1β**: Interleukin-1β; **IL-2**: Interleukin-2; **IL-4**: Interleukin-4; **IL-5**: Interleukin-5; **IL-6**: Interleukin-6; **IL-7**: Interleukin-7; **IL-8**: Interleukin-8; **IL-10**: Interleukin-10; **IL-12**: Interleukin-12; **IL-13**: Interleukin-13; **IL-17**: Interleukin-17; **GCSF**: Granulocyte Colony-Stimulating Factor; **GMCSF**: Granulocyte Monocyte Colony-Stimulating Factor; **INF-γ**: Interferon-γ **MCP-1**: Monocyte Chemoattractant Protein 1; **MIP-1β**: Macrophage Inflammatory Protein 1β; **TNF-α**: Tumor Necrosis Factor-α.

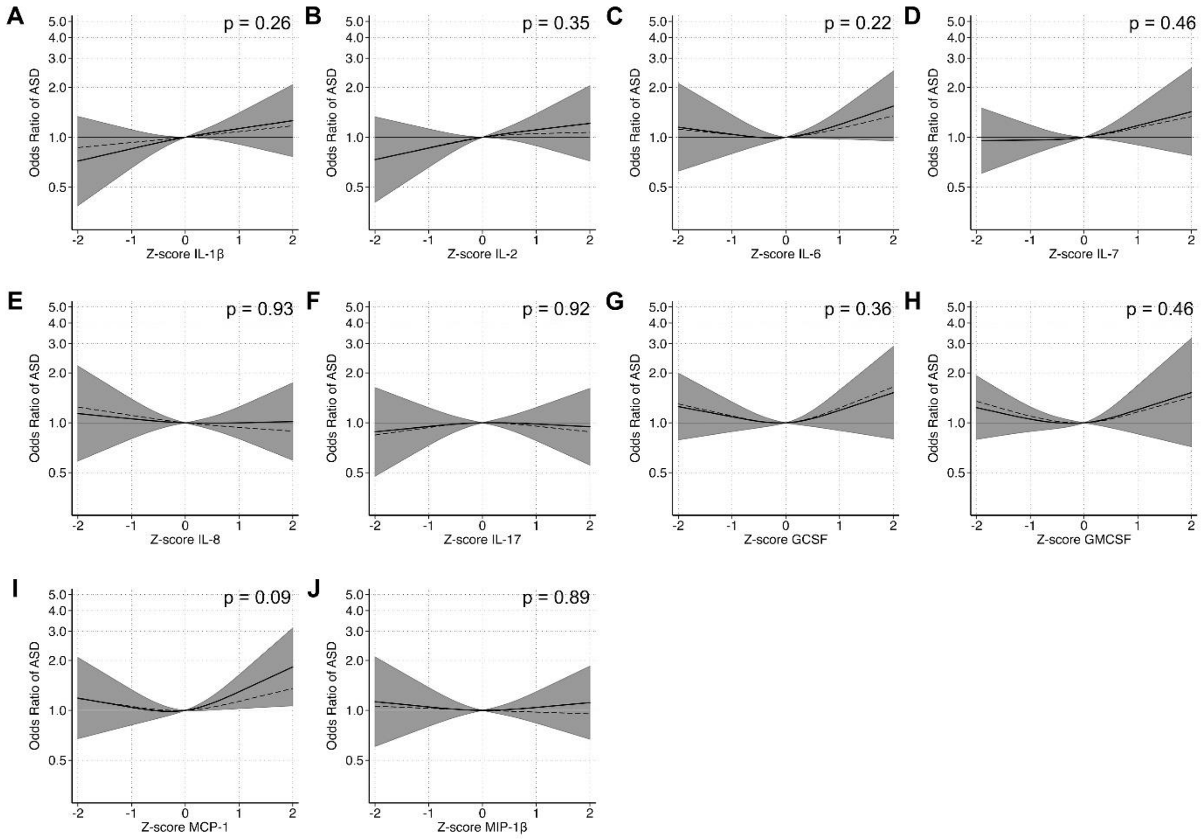

Supplementary Figure 8. The relationship between maternal cytokines and odds of ASD without co-occurring ID or ADHD ("ASD only") when comparing 318 individuals affected by ASD without co-occurring ID or ADHD to 429 unaffected individuals selected from the cohort. Each panel displays the odds of ASD according to cytokine z-scores, flexibly fit using restricted cubic spline models with three knots and a z-score=0 as the referent. The dashed line represents the unadjusted estimate of the relationship between each cytokine and odds of ASD. The solid line represents the fully adjusted model, adjusted for sex of fetus; family income quintile; maternal BMI, psychiatric history, region of origin, and age. The gray bands represent the 95% confidence interval for the fully adjusted model. P-values are shown for a Wald test with a null hypothesis that all cytokine spline terms were jointly equal to zero, as a test of whether each cytokine was generally associated with the outcome. Abbreviations: **IL-1β**: Interleukin-1β; **IL-2**: Interleukin-2; **IL-4**: Interleukin-4; **IL-5**: Interleukin-5; **IL-6**: Interleukin-6; **IL-7**: Interleukin-7; **IL-8**: Interleukin-8; **IL-10**: Interleukin-10; **IL-12**: Interleukin-12; **IL-13**: Interleukin-13; **IL-17**: Interleukin-17; **GCSF**: Granulocyte Colony-Stimulating Factor; **GMCSF**: Granulocyte Monocyte Colony-Stimulating Factor; **INF-γ**: Interferon-γ **MCP-1**: Monocyte Chemoattractant Protein 1; **MIP-1β**: Macrophage Inflammatory Protein 1β; **TNF-α**: Tumor Necrosis Factor-α.

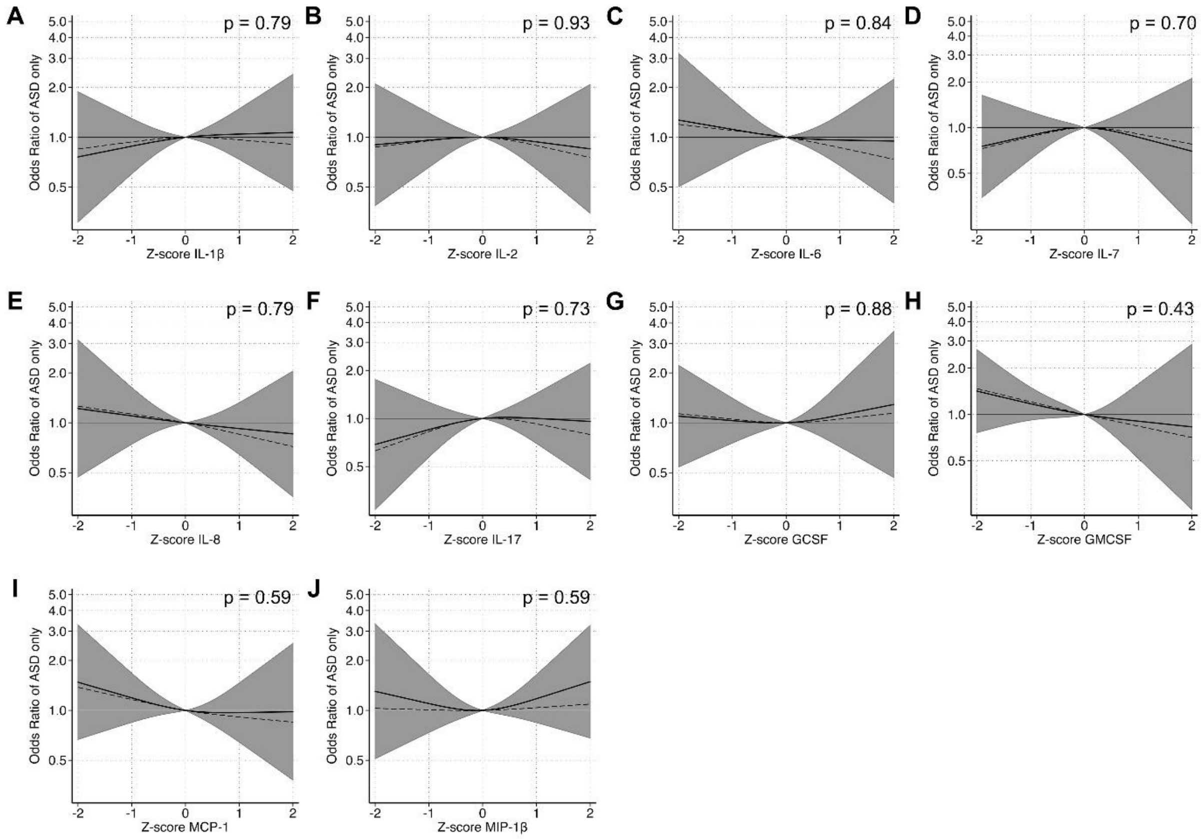

Supplementary Figure 9. The relationship between maternal cytokines and odds of ASD with co-occurring ID when comparing 318 individuals affected by ASD with co-occurring ID to 429 unaffected individuals selected from the cohort. Each panel displays the odds of ASD according to cytokine z-scores, flexibly fit using restricted cubic spline models with three knots and a z-score=0 as the referent. The dashed line represents the unadjusted estimate of the relationship between each cytokine and odds of ASD. The solid line represents the fully adjusted model, adjusted for sex of fetus; family income quintile; maternal BMI, psychiatric history, region of origin, and age. The gray bands represent the 95% confidence interval for the fully adjusted model. P-values are shown for a Wald test with a null hypothesis that all cytokine spline terms were jointly equal to zero, as a test of whether each cytokine was generally associated with the outcome. Abbreviations: **IL-1β**: Interleukin-1β; **IL-2**: Interleukin-2; **IL-4**: Interleukin-4; **IL-5**: Interleukin-5; **IL-6**: Interleukin-6; **IL-7**: Interleukin-7; **IL-8**: Interleukin-8; **IL-10**: Interleukin-10; **IL-12**: Interleukin-12; **IL-13**: Interleukin-13; **IL-17**: Interleukin-17; **GCSF**: Granulocyte Colony-Stimulating Factor; **GMCSF**: Granulocyte Monocyte Colony-Stimulating Factor; **INF-γ**: Interferon-γ **MCP-1**: Monocyte Chemoattractant Protein 1; **MIP-1β**: Macrophage Inflammatory Protein 1β; **TNF-α**: Tumor Necrosis Factor-α.

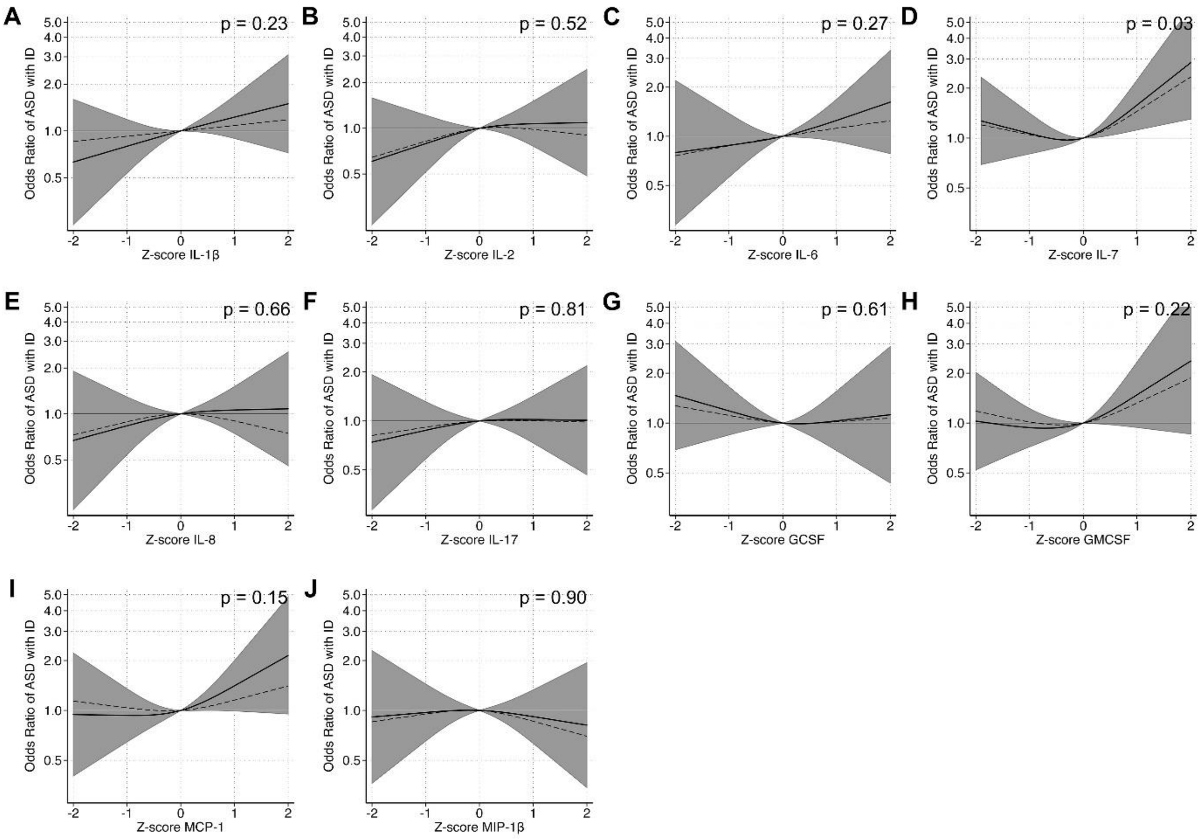

Supplementary Figure 10. The relationship between maternal cytokines and odds of ASD with co-occurring ADHD when comparing 318 individuals affected by ASD with co-occurring ADHD to 429 unaffected individuals selected from the cohort. Each panel displays the odds of ASD according to cytokine z-scores, flexibly fit using restricted cubic spline models with three knots and a z-score=0 as the referent. The dashed line represents the unadjusted estimate of the relationship between each cytokine and odds of ASD. The solid line represents the fully adjusted model, adjusted for sex of fetus; family income quintile; maternal BMI, psychiatric history, region of origin, and age. The gray bands represent the 95% confidence interval for the fully adjusted model. P-values are shown for a Wald test with a null hypothesis that all cytokine spline terms were jointly equal to zero, as a test of whether each cytokine was generally associated with the outcome. Abbreviations: **IL-1β**: Interleukin-1β; **IL-2**: Interleukin-2; **IL-4**: Interleukin-4; **IL-5**: Interleukin-5; **IL-6**: Interleukin-6; **IL-7**: Interleukin-7; **IL-8**: Interleukin-8; **IL-10**: Interleukin-10; **IL-12**: Interleukin-12; **IL-13**: Interleukin-13; **IL-17**: Interleukin-17; **GCSF**: Granulocyte Colony-Stimulating Factor; **GMCSF**: Granulocyte Monocyte Colony-Stimulating Factor; **INF-γ**: Interferon-γ **MCP-1**: Monocyte Chemoattractant Protein 1; **MIP-1β**: Macrophage Inflammatory Protein 1β; **TNF-α**: Tumor Necrosis Factor-α.

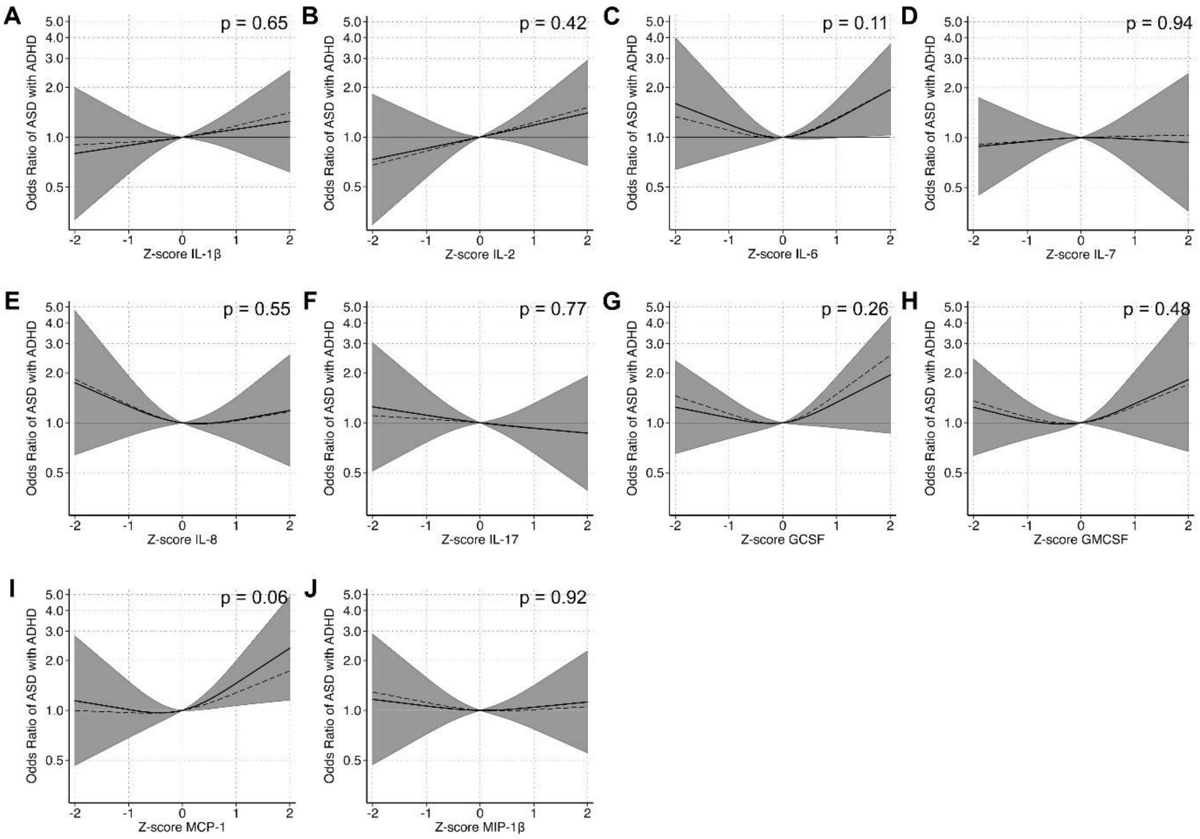

Supplementary Figure 11. The cumulative variation of cytokines and acute phase proteins explained by the principal components.

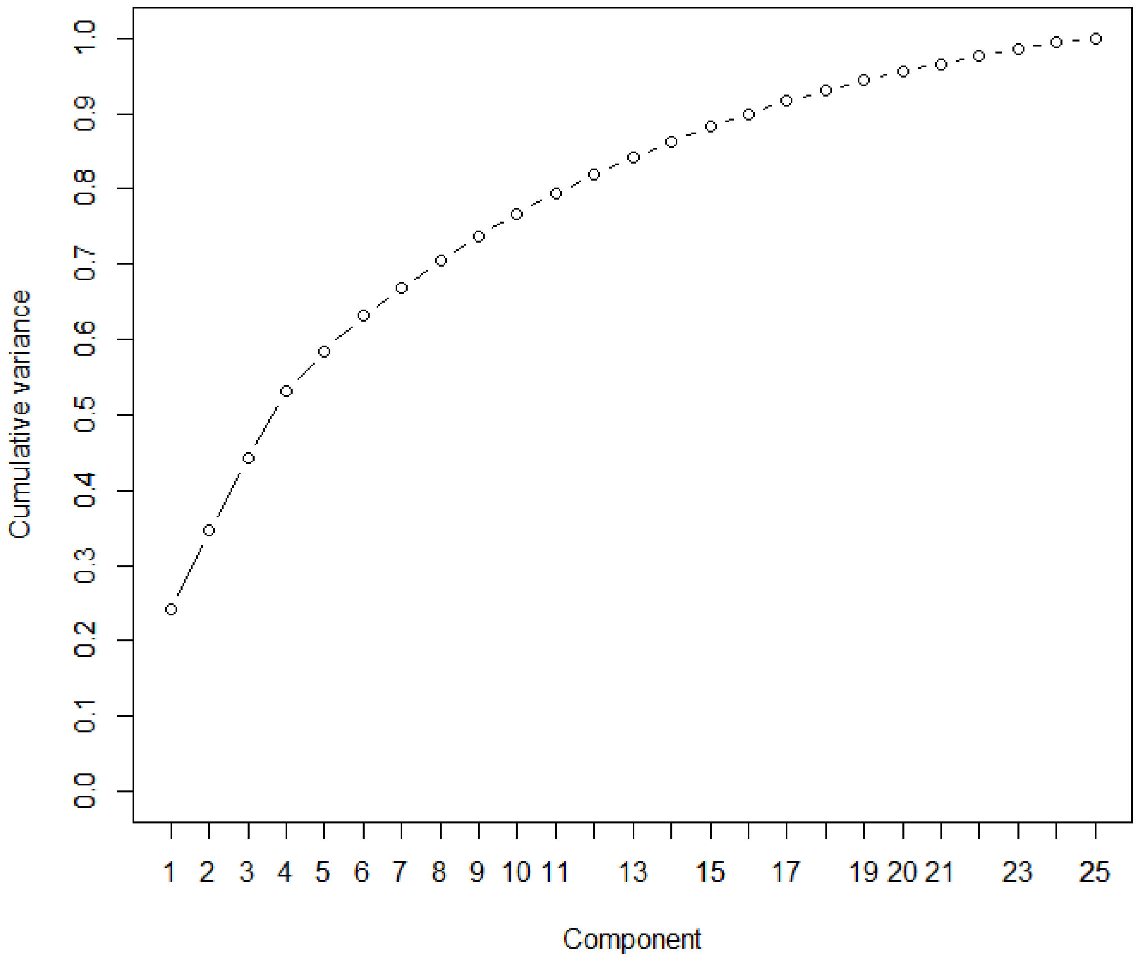

Supplementary Figure 12. The variation in cytokines and acute phase proteins explained by each of the first 10 principal components.

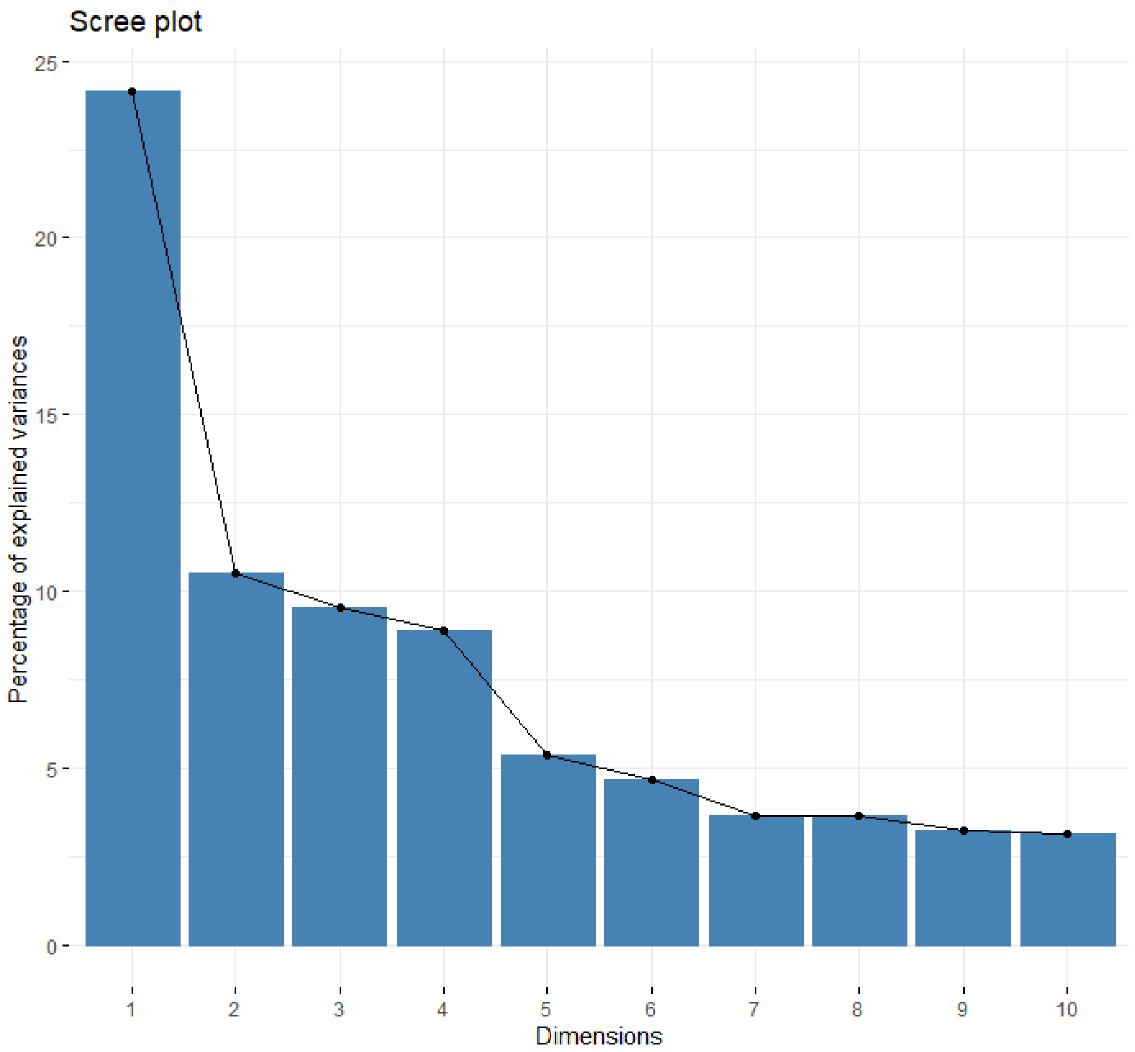

Supplementary Figure 13. Biplot showing the factor loadings by the individual cytokines and acute phase proteins on PC1 and PC3 (**A**), PC1 and PC4 (**B**), PC2 and PC3 (**C**), PC2 and PC4 (**D**), PC3 and PC4 (**E**), and the values of these components for mothers to each individual in the study (n=747, Groups: 0 = unaffected controls (red circles); 1 = ASD-cases (blue triangles)), in the two-dimensional component space generated by each combination of components. Abbreviations: **A2M**: α-2 Macroglobulin; **APP**: Acute Phase Protein; **CRP**: C-Reactive Protein; **Cyto**: Cytokine; **FER**: Ferritin; **FIB**: Fibrinogen; **IL-1β**: Interleukin-1β; **IL-2**: Interleukin-2; **IL-4**: Interleukin-4; **IL-5**: Interleukin-5; **IL-6**: Interleukin-6; **IL-7**: Interleukin-7; **IL-8**: Interleukin-8; **IL-10**: Interleukin-10; **IL-12**: Interleukin-12; **IL-13**: Interleukin-13; **IL-17**: Interleukin-17; **GCSF**: Granulocyte Colony-Stimulating Factor; **GMCSF**: Granulocyte Monocyte Colony-Stimulating Factor; **INF-γ**: Interferon-γ **MCP-1**: Monocyte Chemoattractant Protein 1; **MIP-1β**: Macrophage Inflammatory Protein 1β; **PCT**: Procalcitonin; **SAA**: Serum Amyloid A; Serum Amyloid P; **tPA**: Tissue Plasminogen Activator; **TNF-α**: Tumor Necrosis Factor-α.

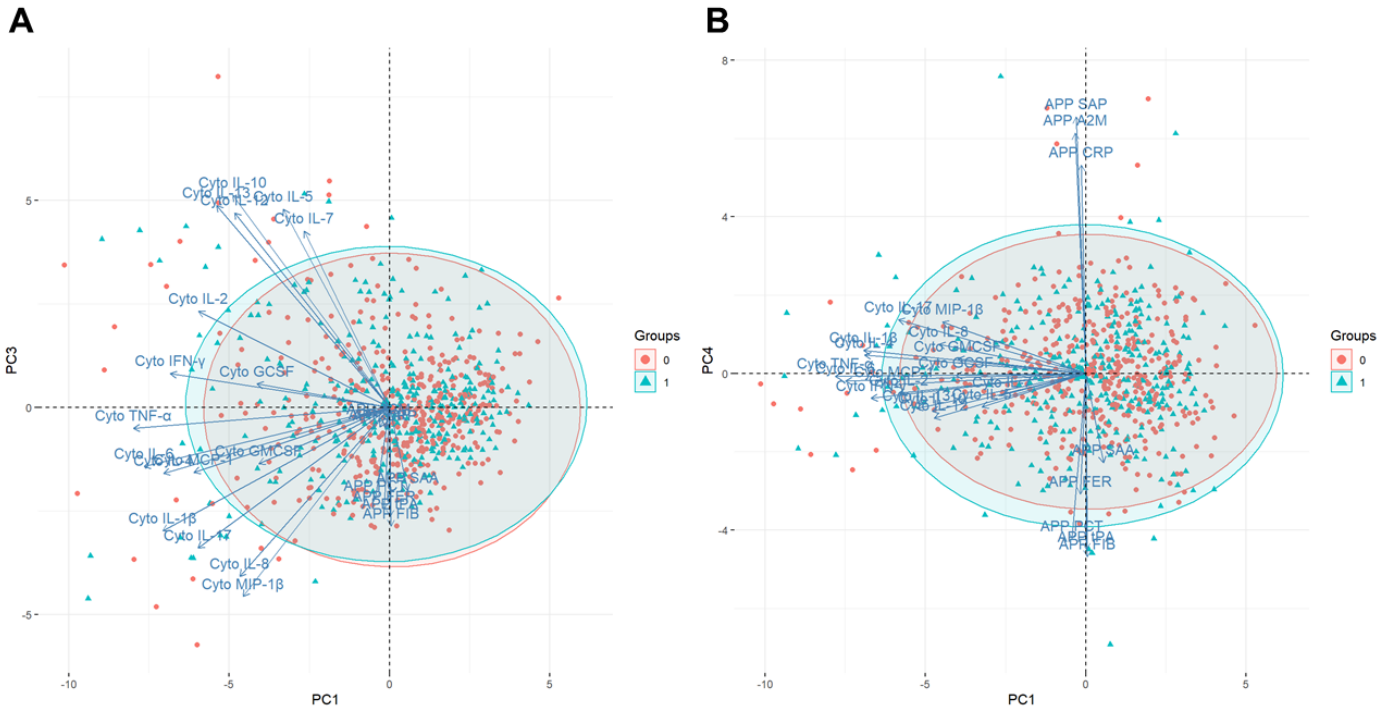

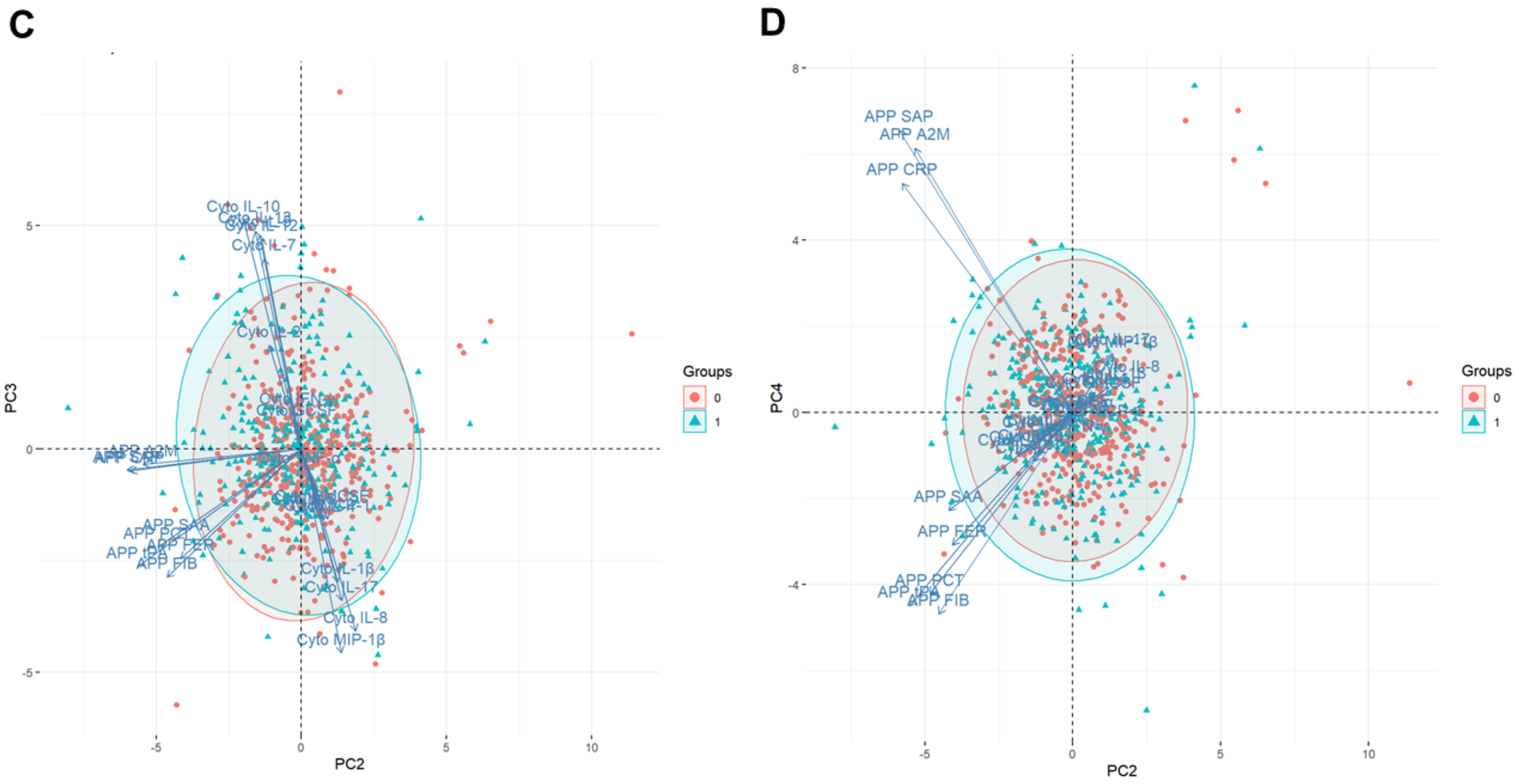
C
D
PC2
PC3
PC4
Groups
0
1
Cyto IL-10
Cyto IL-7
Cyto IL-8
Cyto MIP-1β
Cyto IL-1β
APP SAA
APP FIB
APP FER
APP PCT
APP SAP
APP A2M
APP CRP
5
0
-5
10
8
4
-4

E

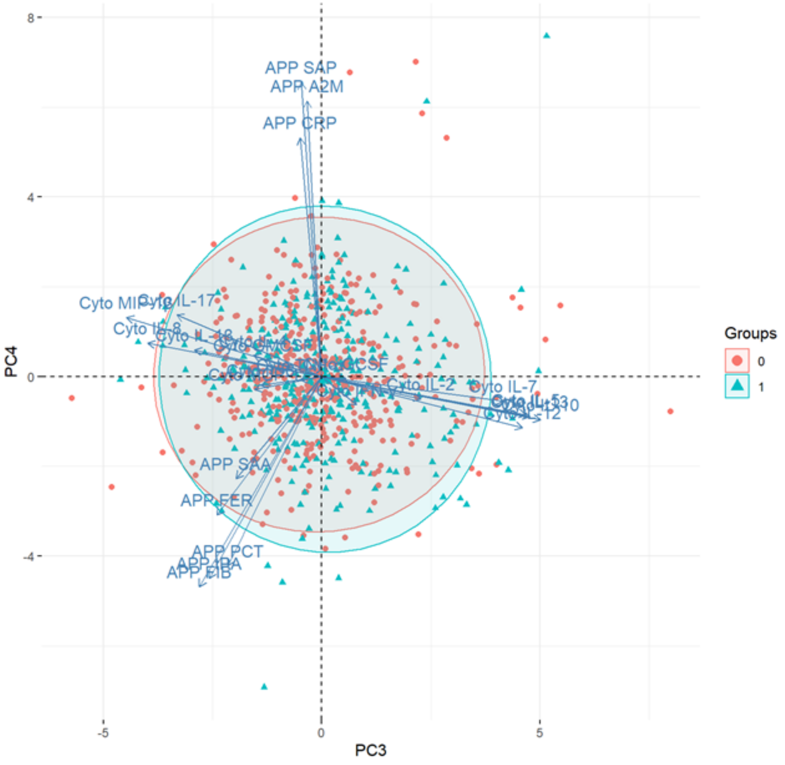

APP SAP
APP A2M
APP CRP
Cyto IL-17
Cyto IL-8
Cyto IL-2
Cyto IL-7
APP SAA
APP FER
APP PCT
APP FIB
PC4
PC3
Groups
0
1

Supplementary Figure 14. Distribution of values of the first four (PC1-PC4) principal components, derived from all cytokines and acute phase proteins, among mothers to the individuals in the study (n=747).

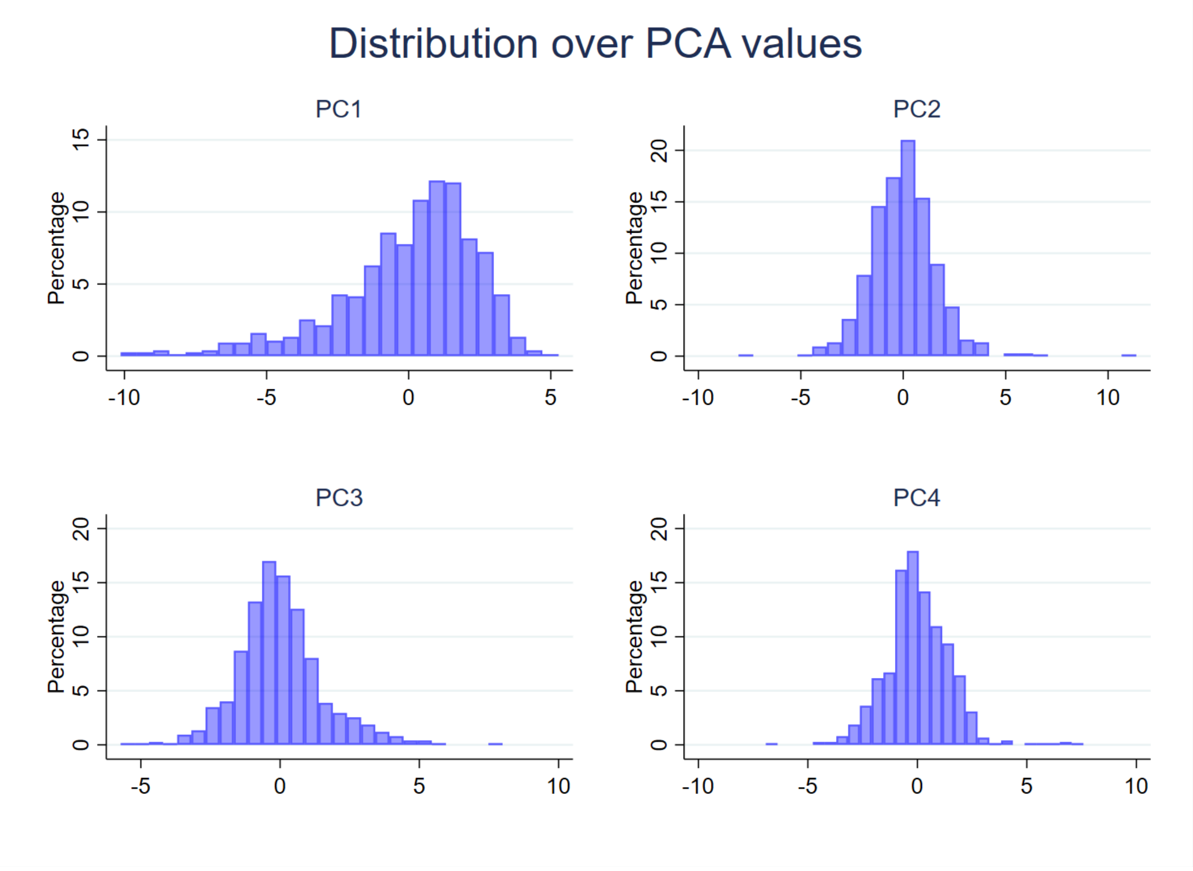

Supplementary Figure 15. The relationship between the first four principal components (PC1-PC4) derived from cytokines and acute phase proteins, and odds of ASD without co-occurring ID or ADHD ("ASD only") when comparing mothers of 318 individuals affected by ASD without co-occurring ID or ADHD to mothers of 429 unaffected individuals selected from the cohort. Each panel displays the odds of ASD according to principal component scores, flexibly fit using restricted cubic spline models with four knots and a score=0 as the referent. The dashed line represents the unadjusted estimate of the relationship between each principal component and odds of ASD. The solid line represents the fully adjusted model, adjusted for sex of fetus; family income quintile; maternal BMI, psychiatric history, region of origin, and age. The gray bands represent the 95% confidence interval for the fully adjusted model. P-values are shown for a Wald test with a null hypothesis that all spline terms were jointly equal to zero, as a test of whether each principal component was generally associated with the outcome. Abbreviations: **PC:** Principal component

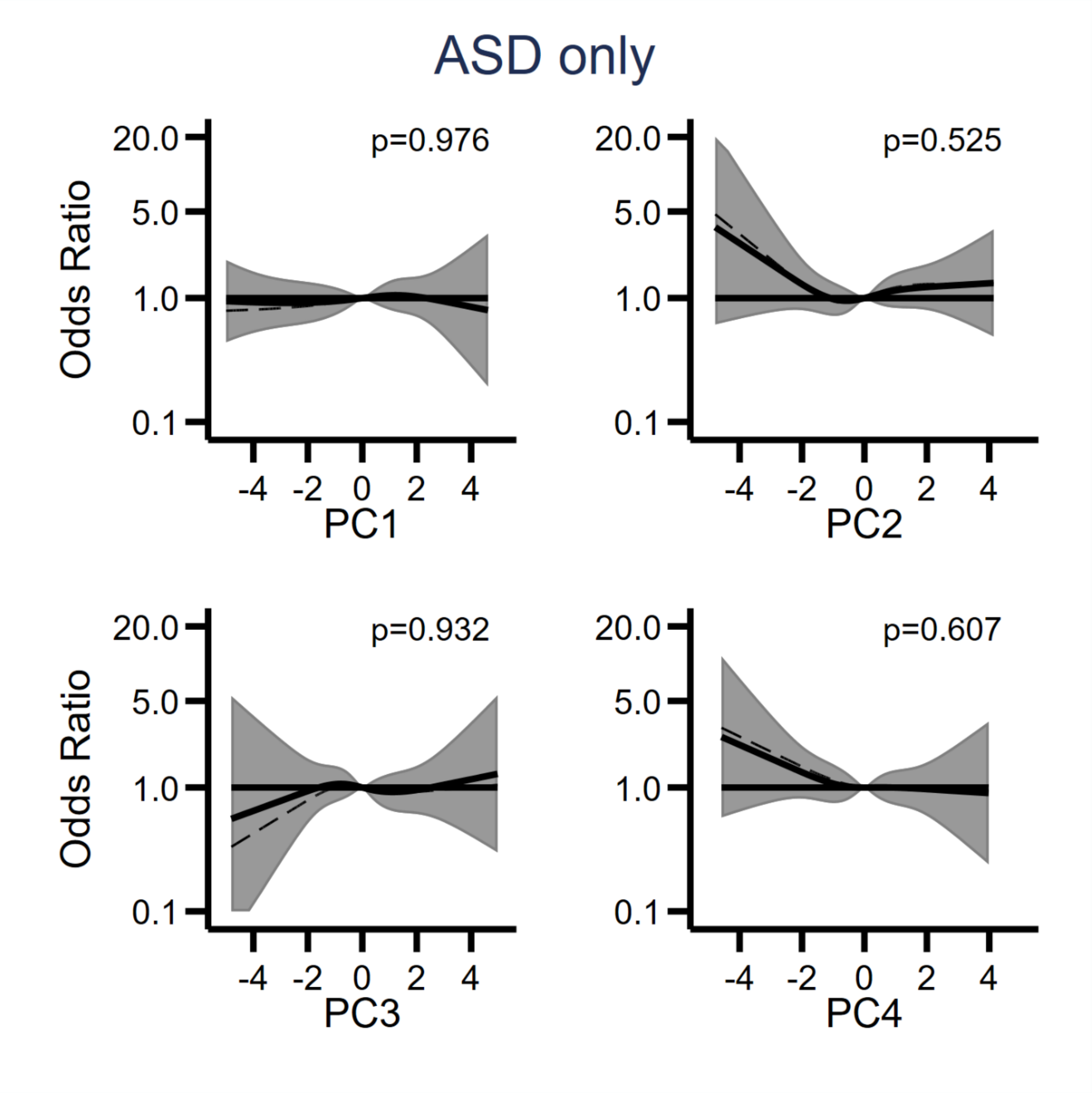

Supplementary Figure 16. The relationship between the first four principal components (PC1-PC4) derived from cytokines and acute phase proteins, and odds of ASD with co-occurring ID when comparing 318 mothers of individuals affected by ASD with co-occurring ID to 429 mothers of unaffected individuals selected from the cohort. Each panel displays the odds of ASD according to principal component scores, flexibly fit using restricted cubic spline models with four knots and a score=0 as the referent. The dashed line represents the unadjusted estimate of the relationship between each principal component and odds of ASD. The solid line represents the fully adjusted model, adjusted for sex of fetus; family income quintile; maternal BMI, psychiatric history, region of origin, and age. The gray bands represent the 95% confidence interval for the fully adjusted model. P-values are shown for a Wald test with a null hypothesis that all spline terms were jointly equal to zero, as a test of whether each principal component was generally associated with the outcome. Abbreviations: **PC:** Principal component

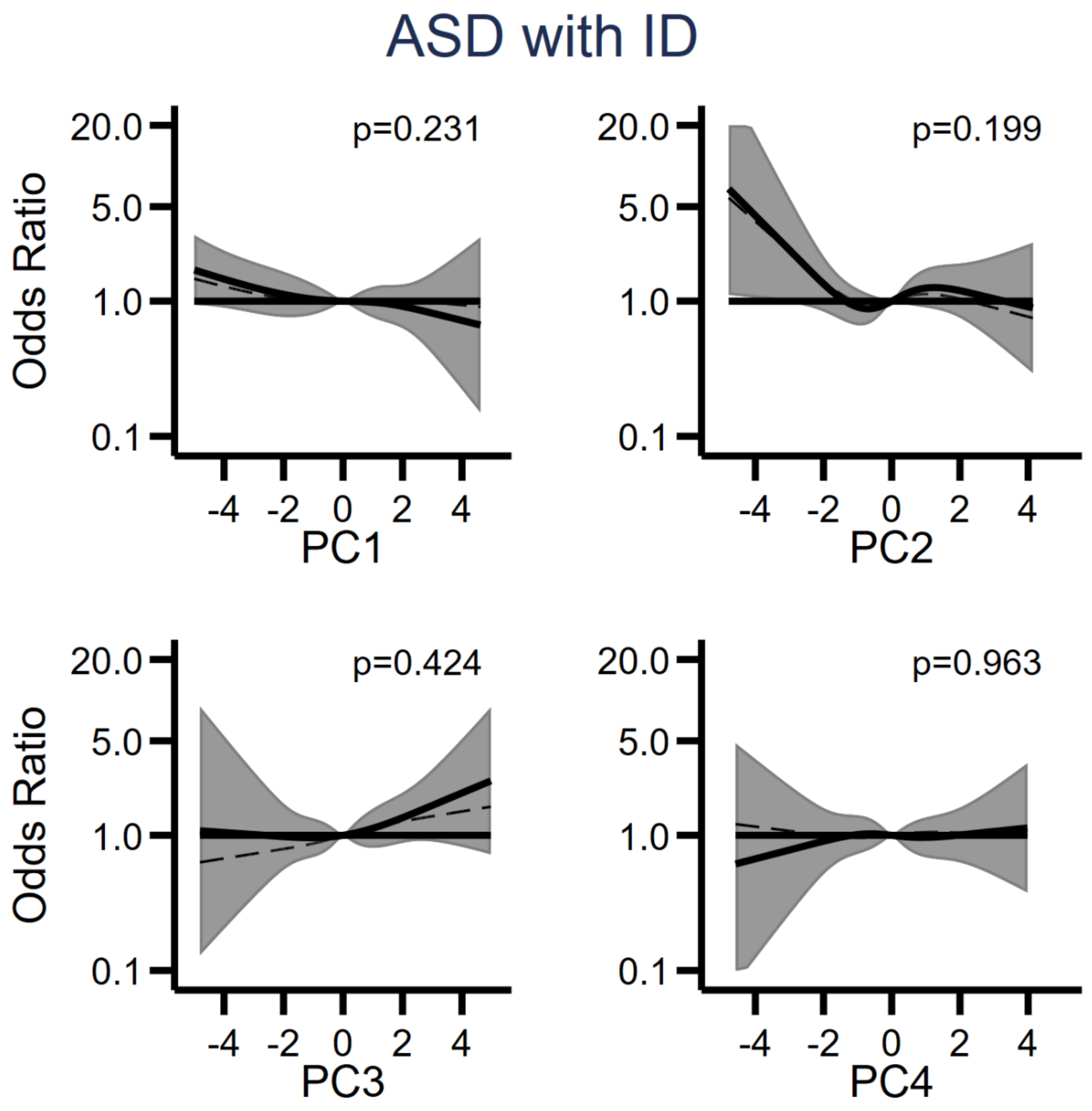

Supplementary Figure 17. The relationship between the first four principal components (PC1-PC4) derived from cytokines and acute phase proteins, and odds of ASD with co-occurring ADHD when comparing 318 mothers of individuals affected by ASD with co-occurring ADHD to 429 mothers of unaffected individuals selected from the cohort. Each panel displays the odds of ASD according to principal component scores, flexibly fit using restricted cubic spline models with four knots and a score=0 as the referent. The dashed line represents the unadjusted estimate of the relationship between each principal component and odds of ASD. The solid line represents the fully adjusted model, adjusted for sex of fetus; family income quintile; maternal BMI, psychiatric history, region of origin, and age. The gray bands represent the 95% confidence interval for the fully adjusted model. P-values are shown for a Wald test with a null hypothesis that all spline terms were jointly equal to zero, as a test of whether each principal component was generally associated with the outcome.
Abbreviations: **PC:** Principal component

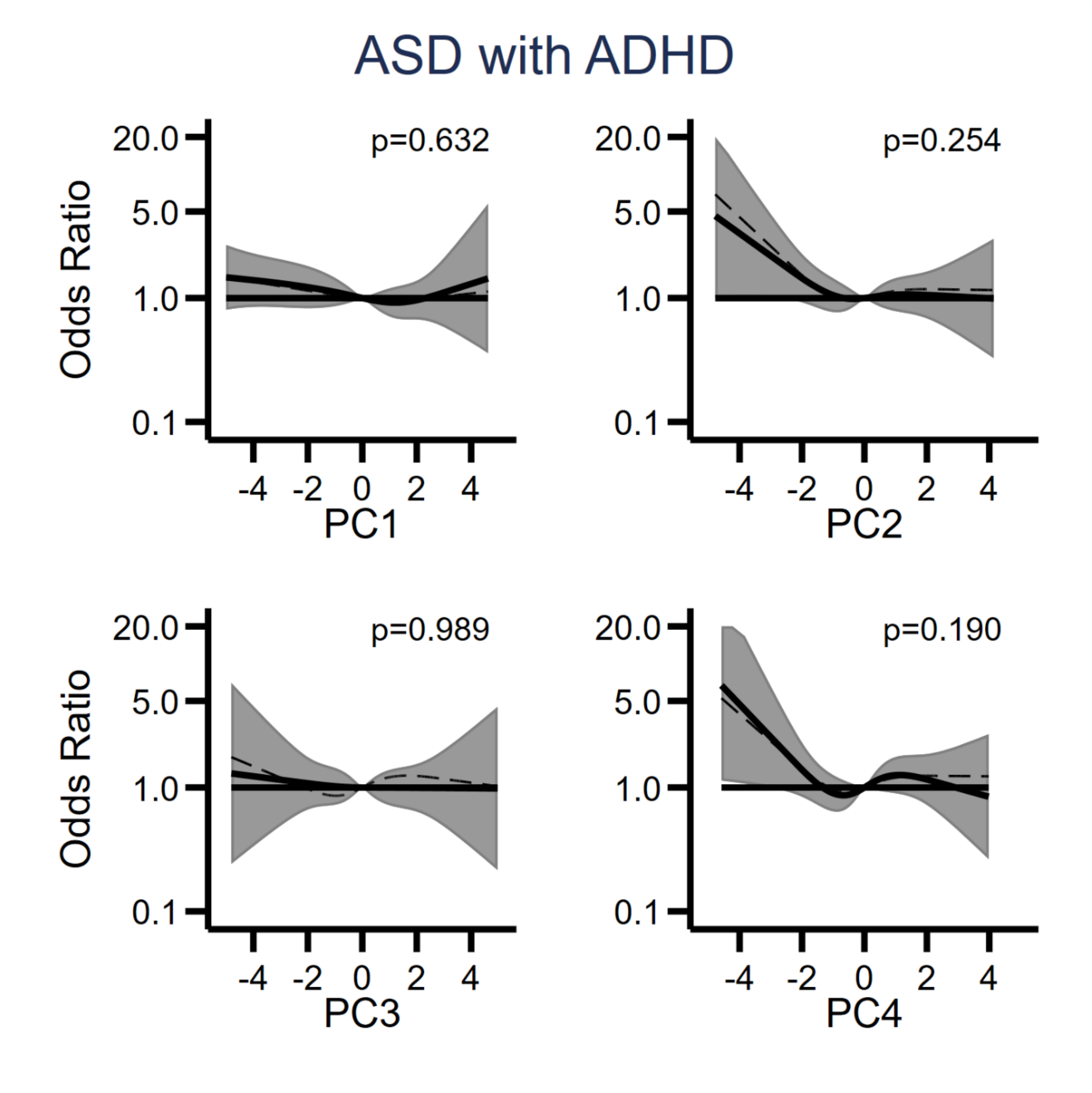

Supplementary Figure 18. The relationship between the first four principal components (PC1-PC4) derived from cytokines and acute phase proteins, and odds of ASD when comparing 430 individuals affected by ASD to 549 unaffected individuals selected from the cohort, when samples from all trimesters were included as a sensitivity analysis. Each panel displays the odds of ASD according to principal component scores, flexibly fit using restricted cubic spline models with four knots and a score=0 as the referent. The dashed line represents the unadjusted estimate of the relationship between each principal component and odds of ASD. The solid line represents the fully adjusted model, adjusted for sex of fetus; family income quintile; maternal BMI, psychiatric history, region of origin, and age. The gray bands represent the 95% confidence interval for the fully adjusted model. P-values are shown for a Wald test with a null hypothesis that all spline terms were jointly equal to zero, as a test of whether each principal component was generally associated with the outcome. Abbreviations: **PC:** Principal Component.

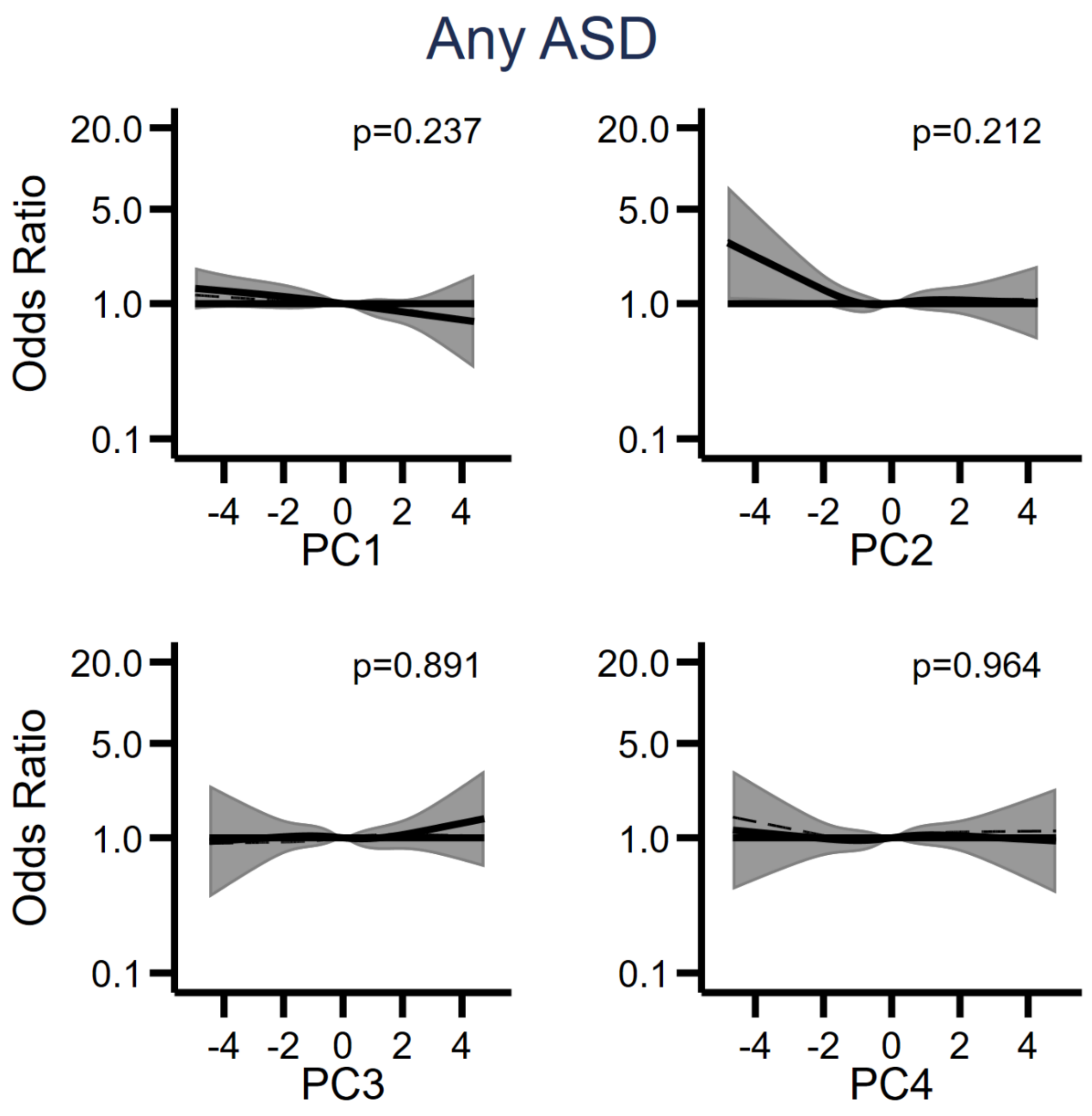

1.2 Supplementary Tables

Supplementary Table 1. Quality control statistics for multiplex assays to analyze cytokine concentrations in maternal serum samples.

Analyte	Inter-assay %CV (controls)[a]	Intra-assay %CV (controls)[a]	Below LLOQ	LLOQ[b]	Above ULOQ	ULOQ[c]	Imputed[d]
Interleukin 1β	16.7	6.66	7 (0.94%)	0.5 pg/ml	0 (0.00%)	3109.65 pg/ml	336 (44.98%)
Interleukin 2	22.2	4.83	11 (1.47%)	1.02 pg/ml	0 (0.00%)	11678.4 pg/ml	253 (33.87%)
Interleukin 4	21.3	4.89	314 (42.03%)	0.27 pg/ml	0 (0.00%)	2011.49 pg/ml	672 (89.96%)
Interleukin 5	17.4	6.23	635 (85.01%)	1.38 pg/ml	0 (0.00%)	5650.8 pg/ml	697 (93.31%)
Interleukin 6	22.1	6.13	12 (1.61%)	2.31 pg/ml	0 (0.00%)	9101.84 pg/ml	479 (64.12%)
Interleukin 7	21.0	9.06	13 (1.74%)	0.91 pg/ml	0 (0.00%)	5872.37 pg/ml	127 (17.00%)
Interleukin 8	22.5	6.17	5 (0.67%)	2.63 pg/ml	9 (1.2%)	6087.59 pg/ml	141 (18.88%)
Interleukin 10	18.7	6.2	1 (0.13%)	2.14 pg/ml	0 (0.00%)	7776.53 pg/ml	592 (79.25%)
Interleukin 12	23.3	9.24	186 (24.90%)	2.33 pg/ml	0 (0.00%)	18204.58 pg/ml	580 (77.64%)
Interleukin 13	19.6	3.61	471 (63.05%)	0.7 pg/ml	0 (0.00%)	7952.08 pg/ml	543 (72.69%)
Interleukin 17	23.2	5.35	3 (0.40%)	1.75 pg/ml	0 (0.00%)	22817.43 pg/ml	62 (8.30%)

Granulocyte Colony-Stimulating Factor	22.3	6.11	10 (1.34%)	2.22 pg/ml	0 (0.00%)	16889.82 pg/ml	397 (53.15%)
Granulocyte Monocyte Colony-Stimulating Factor	19.2	2.49	109 (14.59%)	1.55 pg/ml	0 (0.00%)	13502.37 pg/ml	171 (22.89%)
Interferon γ	34.9	7.43	413 (55.29%)	5.5 pg/ml	0 (0.00%)	7102.16 pg/ml	564 (75.50%)
Monocyte Chemoattractant Protein 1	22.3	7.46	9 (1.20%)	1.46 pg/ml	0 (0.00%)	5917.82 pg/ml	20 (2.68%)
Macrophage Inflammatory Protein 1β	18.5	6.73	0 (0.00%)	0.56 pg/ml	0 (0.00%)	2096.68 pg/ml	0 (0.00%)
Tumor Necrosis Factor α	15.1	5.01	49 (6.56%)	3.9 pg/ml	0 (0.00%)	41506.33 pg/ml	637 (85.27%)
Average	21.2	6.09	18.81%		0.53%		49.38%

a. The percent coefficients of variation (CV) is based on standardized controls that were run in duplicate on each of the 13 assay plates.
b. Lower Limit of Quantitation, average across 13 assay plates.
c. Upper Limit of Quantitation, average across 13 assay plates.
d. The absolute and relative frequency of observations that were imputed, including those values that were below the LLOQ, above the ULOQ, or that the Bio-plex Manager software indicated were near the LLOQ and thus less certain than values higher in the range of quantitation.

Supplementary Table 2. P-values and β-coefficients for the linear relationship between cytokines and gestational age at serum sampling.

	Full sample (n=979)		***First trimester (n=747)***		***Later trimesters (n=232)***	
	β-coeff.	P-value	β-coeff.	P-value	β-coeff.	P-value
IL-1β	-0.009	0.180	0.019	0.380	-0.014	0.164
IL-2	-0.008	0.203	0.038	0.075	-0.016	0.133
IL-4	-0.009	0.151	0.005	0.826	-0.007	0.522
IL-5	<0.001	0.941	0.015	0.484	-0.001	0.950
IL-6	<0.001	0.958	0.047	**0.038**	-0.009	0.353
IL-7	0.009	0.183	0.016	0.472	0.014	0.186
IL-8	-0.014	**0.036**	0.006	0.803	-0.016	0.127
IL-10	0.001	0.847	0.010	0.637	0.004	0.680
IL-12	-0.006	0.358	-0.014	0.525	-0.009	0.408
IL-13	0.011	0.085	0.023	0.297	0.017	0.101
IL-17	-0.014	**0.028**	-0.016	0.468	-0.011	0.282
GCSF	0.021	**0.001**	0.010	0.638	0.016	0.102
GMCSF	0.003	0.614	0.002	0.922	0.004	0.713
INF-γ	-0.009	0.154	0.017	0.455	-0.007	0.522
MCP-1	-0.022	**0.001**	0.027	0.226	-0.021	0.057
MIP-1β	-0.022	**0.001**	-0.014	0.544	-0.020	**0.049**
TNF-α	-0.004	0.522	0.018	0.392	-0.006	0.549

Abbreviations: **Coeff**: Coefficient; **IL-1β**: Interleukin 1β; **IL-2**: Interleukin 2; **IL-4**: Interleukin 4; **IL-6**: Interleukin 6; **IL-7**: Interleukin 7; **IL-8**: Interleukin 8; **IL-10**: Interleukin 10; **IL-12**: Interleukin 12; **IL-13**: Interleukin 13; **IL-17**: Interleukin-17; **GCSF**: Granulocyte Colony-Stimulating Factor; **GMCSF**: Granulocyte Monocyte Colony-Stimulating Factor; **INF-γ**: Interferon γ; **MCP-1**: Monocyte Chemoattractant Protein 1; **MIP-1β**: Macrophage Inflammatory Protein 1β; **TNF-α**: Tumor Necrosis Factor α.

Supplementary Table 3. The unadjusted relationship between cytokines and odds of ASD, stratified by co-occurrence of ID and ADHD, when comparing mothers of 318 ASD-cases to mothers of 429 unaffected individuals selected from the cohort. Dichotomous variables were created for each cytokine, using the distribution of z-scores among unaffected individuals to set the cut-offs, using values below the 90th percentile as the referent category. Models were adjusted for sex, maternal BMI, maternal psychiatric history, maternal region of origin, maternal age and family income quintile. P-values are shown for a Wald test with a null hypothesis that the cytokine categorical term was equal to zero, as a test of whether each cytokine was associated with the outcome. Abbreviations: **IL-1β**: Interleukin-1β; **IL-2**: Interleukin-2; **IL-4**: Interleukin-4; **IL-5**: Interleukin-5; **IL-6**: Interleukin-6; **IL-7**: Interleukin-7; **IL-8**: Interleukin-8; **IL-10**: Interleukin-10; **IL-12**: Interleukin-12; **IL-13**: Interleukin-13; **IL-17**: Interleukin-17; **GCSF**: Granulocyte Colony-Stimulating Factor; **GMCSF**: Granulocyte Monocyte Colony-Stimulating Factor; **INF-γ**: Interferon-γ **MCP-1**: Monocyte Chemoattractant Protein 1; **MIP-1β**: Macrophage Inflammatory Protein 1β; **TNF-α**: Tumor Necrosis Factor-α.

	**Any ASD (OR [LCI; UCI])**	**p-value**	**ASD only (OR [LCI; UCI])**	**p-value**	**ASD with ID (OR [LCI; UCI])**	**p-value**	**ASD with ADHD (OR [LCI; UCI])**	**p-value**
IL-1β	1.52 (0.97; 2.37)	0.065	1.22 (0.62; 2.42)	0.560	1.69 (0.91; 3.14)	0.097	1.63 (0.9; 2.95)	0.105
IL-2	1.22 (0.77; 1.94)	0.403	1.00 (0.48; 2.06)	0.994	0.99 (0.48; 2.04)	0.971	1.63 (0.9; 2.95)	0.105
IL-4	1.22 (0.77; 1.93)	0.389	0.87 (0.41; 1.84)	0.707	1.41 (0.74; 2.68)	0.298	1.39 (0.75; 2.56)	0.296
IL-5	1.08 (0.68; 1.73)	0.742	0.87 (0.41; 1.84)	0.707	1.65 (0.89; 3.06)	0.114	0.82 (0.4; 1.68)	0.584
IL-6	1.48 (0.95; 2.31)	0.085	1.11 (0.55; 2.24)	0.772	1.57 (0.83; 2.95)	0.165	1.74 (0.97; 3.12)	0.063
IL-7	1.22 (0.77; 1.94)	0.403	0.89 (0.42; 1.89)	0.757	**2.08 (1.15; 3.75)**	**0.015**	0.84 (0.41; 1.72)	0.633
IL-8	0.94	0.789	0.68	0.355	0.67	0.342	1.42	0.261

	(0.57; 1.53)		(0.29; 1.55)		(0.29; 1.53)		(0.77; 2.63)	
IL-10	**1.56 (1; 2.42)**	**0.050**	1.34 (0.69; 2.6)	0.385	**1.95 (1.07; 3.54)**	**0.029**	1.42 (0.77; 2.63)	0.261
IL-12	1.52 (0.97; 2.37)	0.065	1.34 (0.69; 2.6)	0.385	1.44 (0.76; 2.76)	0.265	1.74 (0.97; 3.12)	0.063
IL-13	1.48 (0.95; 2.31)	0.085	1.22 (0.62; 2.42)	0.560	**2.5 (1.42; 4.41)**	**0.002**	0.93 (0.46; 1.87)	0.842
IL-17	1.11 (0.69; 1.78)	0.664	1.11 (0.55; 2.24)	0.772	1.33 (0.68; 2.57)	0.403	0.93 (0.46; 1.87)	0.842
GCSF	1.18 (0.74; 1.88)	0.482	1.00 (0.48; 2.06)	0.994	0.99 (0.48; 2.04)	0.971	1.53 (0.83; 2.79)	0.170
GMCSF	1.22 (0.77; 1.93)	0.389	0.76 (0.35; 1.67)	0.496	1.29 (0.67; 2.5)	0.446	1.59 (0.88; 2.87)	0.124
INF-γ	1.25 (0.79; 1.99)	0.333	0.78 (0.35; 1.72)	0.538	1.21 (0.61; 2.39)	0.582	1.74 (0.97; 3.12)	0.063
MCP-1	1.07 (0.67; 1.73)	0.766	0.68 (0.29; 1.55)	0.355	1.44 (0.76; 2.76)	0.265	1.12 (0.58; 2.16)	0.731
MIP-1β	1.07 (0.67; 1.73)	0.766	1.34 (0.69; 2.6)	0.385	0.77 (0.35; 1.7)	0.520	1.12 (0.58; 2.16)	0.731
TNF-α	1.48 (0.95; 2.31)	0.085	1.11 (0.55; 2.24)	0.772	1.33 (0.68; 2.57)	0.403	**1.96 (1.11; 3.46)**	**0.020**

Supplementary Table 4. The adjusted relationship between cytokines and odds of ASD, stratified by co-occurrence of ID and ADHD, when comparing 430 ASD cases to 549 unaffected individuals selected from the cohort. As a sensitivity analysis, samples from all trimesters were used. Dichotomous variables were created for each cytokine, using the distribution of z-scores among unaffected individuals to set the cut-offs, using values below the 90th percentile as the referent category. Models were adjusted for sex, maternal BMI, maternal psychiatric history, maternal region of origin, maternal age and family income quintile. P-values are shown for a Wald test with a null hypothesis that the cytokine categorical term was equal to zero, as a test of whether each cytokine was associated with the outcome. Abbreviations: **IL-1β**: Interleukin-1β; **IL-2**: Interleukin-2; **IL-4**: Interleukin-4; **IL-5**: Interleukin-5; **IL-6**: Interleukin-6; **IL-7**: Interleukin-7; **IL-8**: Interleukin-8; **IL-10**: Interleukin-10; **IL-12**: Interleukin-12; **IL-13**: Interleukin-13; **IL-17**: Interleukin-17; **GCSF**: Granulocyte Colony-Stimulating Factor; **GMCSF**: Granulocyte Monocyte Colony-Stimulating Factor; **INF-γ**: Interferon-γ **MCP-1**: Monocyte Chemoattractant Protein 1; **MIP-1β**: Macrophage Inflammatory Protein 1β; **TNF-α**: Tumor Necrosis Factor-α.

	**Any ASD (OR [LCI; UCI])**	**p-value**	**ASD only (OR [LCI; UCI])**	**p-value**	**ASD with ID (OR [LCI; UCI])**	**p-value**	**ASD with ADHD (OR [LCI; UCI])**	**p-value**
IL-1β	**1.60 (1.05; 2.44)**	0.030	1.38 (0.72; 2.64)	0.336	**2.07 (1.15; 3.71)**	0.015	1.54 (0.83; 2.83)	0.169
IL-2	1.20 (0.77; 1.87)	0.428	0.93 (0.46; 1.87)	0.835	1.07 (0.54; 2.1)	0.847	1.37 (0.74; 2.54)	0.317
IL-4	1.23 (0.79; 1.91)	0.353	0.98 (0.50; 1.95)	0.965	1.26 (0.68; 2.34)	0.465	1.17 (0.62; 2.19)	0.632
IL-5	1.00 (0.64; 1.57)	0.983	0.89 (0.45; 1.77)	0.743	1.48 (0.82; 2.68)	0.194	0.64 (0.31; 1.3)	0.214
IL-6	**1.68 (1.10; 2.57)**	0.015	1.55 (0.82; 2.91)	0.176	1.62 (0.89; 2.95)	0.117	**1.97 (1.08; 3.57)**	0.026
IL-7	1.08 (0.69; 1.69)	0.728	0.81 (0.40; 1.65)	0.568	1.72 (0.97; 3.05)	0.064	0.58 (0.27; 1.22)	0.151
IL-8	1.14 (0.72; 1.8)	0.573	1.16	0.673	0.84	0.656	1.61 (0.84; 3.05)	0.148

			(0.59; 2.28)		(0.40; 1.78)			
IL-10	**1.64 (1.08; 2.49)**	0.022	1.49 (0.79; 2.82)	0.223	**2.30 (1.31; 4.01)**	0.004	1.38 (0.72; 2.64)	0.327
IL-12	**1.59 (1.04; 2.43)**	0.031	1.69 (0.90; 3.17)	0.104	1.64 (0.90; 2.99)	0.106	1.73 (0.95; 3.17)	0.075
IL-13	1.25 (0.82; 1.91)	0.305	1.19 (0.63; 2.25)	0.582	**1.82 (1.05; 3.16)**	0.034	0.75 (0.37; 1.53)	0.430
IL-17	1.12 (0.72; 1.74)	0.608	1.31 (0.68; 2.49)	0.418	1.29 (0.70; 2.37)	0.412	0.69 (0.34; 1.41)	0.310
GCSF	1.14 (0.73; 1.76)	0.569	0.91 (0.45; 1.85)	0.791	0.98 (0.52; 1.84)	0.943	1.36 (0.74; 2.52)	0.326
GMCSF	1.09 (0.69; 1.70)	0.722	0.77 (0.36; 1.63)	0.495	0.93 (0.47; 1.83)	0.823	1.73 (0.94; 3.2)	0.079
INF-γ	1.29 (0.83; 2.00)	0.261	0.98 (0.48; 1.99)	0.953	1.21 (0.64; 2.27)	0.559	1.34 (0.72; 2.5)	0.362
MCP-1	1.13 (0.71; 1.79)	0.605	0.68 (0.30; 1.53)	0.349	1.42 (0.74; 2.72)	0.296	1.38 (0.71; 2.65)	0.341
MIP-1β	1.25 (0.80; 1.95)	0.334	1.48 (0.79; 2.79)	0.221	1.21 (0.64; 2.29)	0.554	0.97 (0.49; 1.93)	0.928
TNF-α	**1.58 (1.03; 2.43)**	0.035	1.57 (0.82; 2.99)	0.171	1.53 (0.83; 2.82)	0.176	**1.86 (1; 3.43)**	0.048
